# RNA localization is a key determinant of neurite-enriched proteome

**DOI:** 10.1038/s41467-017-00690-6

**Published:** 2017-09-19

**Authors:** Alessandra Zappulo, David van den Bruck, Camilla Ciolli Mattioli, Vedran Franke, Koshi Imami, Erik McShane, Mireia Moreno-Estelles, Lorenzo Calviello, Andrei Filipchyk, Esteban Peguero-Sanchez, Thomas Müller, Andrew Woehler, Carmen Birchmeier, Enrique Merino, Nikolaus Rajewsky, Uwe Ohler, Esteban O. Mazzoni, Matthias Selbach, Altuna Akalin, Marina Chekulaeva

**Affiliations:** 1 Non-coding RNAs and Mechanisms of Cytoplasmic Gene Regulation, Berlin Institute for Medical Systems Biology, Max Delbrück Center for Molecular Medicine, 13125 Berlin, Germany; 2 BIMSB Bioinformatics Platform, Max Delbrück Center for Molecular Medicine, 13125 Berlin, Germany; 3 Proteome Dynamics, Max Delbrück Center for Molecular Medicine, 13125 Berlin, Germany; 4 Department of Biology, New York University, New York, NY 10003-6688 USA; 5 Computational Regulatory Genomics, Berlin Institute for Medical Systems Biology, Max Delbrück Center for Molecular Medicine, 13125 Berlin, Germany; 6 Systems Biology of Gene Regulatory Elements, Berlin Institute for Medical Systems Biology, Max Delbrück Center for Molecular Medicine, 13125 Berlin, Germany; 7 Departamento de Microbiología Molecular, Instituto de Biotecnología, UNAM, Av. Universidad 2001, Cuernavaca, Morelos, CP 62210 Mexico; 8 Developmental Biology/Signal Transduction, Max Delbrück Center for Molecular Medicine, 13125 Berlin, Germany; 9 BIMSB Light Microscopy Platform, Max Delbrück Center for Molecular Medicine, 13125 Berlin, Germany

## Abstract

Protein subcellular localization is fundamental to the establishment of the body axis, cell migration, synaptic plasticity, and a vast range of other biological processes. Protein localization occurs through three mechanisms: protein transport, mRNA localization, and local translation. However, the relative contribution of each process to neuronal polarity remains unknown. Using neurons differentiated from mouse embryonic stem cells, we analyze protein and RNA expression and translation rates in isolated cell bodies and neurites genome-wide. We quantify 7323 proteins and the entire transcriptome, and identify hundreds of neurite-localized proteins and locally translated mRNAs. Our results demonstrate that mRNA localization is the primary mechanism for protein localization in neurites that may account for half of the neurite-localized proteome. Moreover, we identify multiple neurite-targeted non-coding RNAs and RNA-binding proteins with potential regulatory roles. These results provide further insight into the mechanisms underlying the establishment of neuronal polarity.

## Abstract (web summary)

Subcellular localization of RNAs and proteins is important for polarized cells such as neurons. Here the authors differentiate mouse embryonic stem cells into neurons, and analyze the local transcriptome, proteome, and translated transcriptome in their cell bodies and neurites, providing a unique resource for future studies on neuronal polarity.

## Introduction

Targeting RNAs and proteins to specific cellular compartments has emerged as a powerful and widespread mechanism to establish cellular asymmetry (reviewed in ref. ^1^). Subcellular localization is particularly important for highly polarized cells such as oocytes, migrating cells, and neurons. For example, studies suggest that neuronal extensions, such as neurites (axons and dendrites) can function autonomously at long distances from the cell body largely due to the localization and local translation of messenger RNAs (reviewed in ref. ^1^). Recent high-throughput analyses revealed that mRNA localization affects a large number of mRNAs^2–11^, such that ~20% of mRNAs in the *Drosophila* oocyte show specific localization patterns^12^.

Several mechanisms could explain mRNA localization: (a) directed transport of mRNAs by motor proteins along the cytoskeleton; (b) diffusion combined with entrapment by a prelocalized anchoring protein; and (c) localization-dependent mRNA degradation (reviewed in ref. ^13^). *cis*-Regulatory elements present in the localized mRNAs (zip codes) mediate the specific localization patterns in each mechanism. These *cis-*elements are bound by specific *trans*-acting factors, RNA-binding proteins (RBPs). RBPs can control both mRNA localization by binding to motor proteins or anchoring proteins and repress mRNA translation before reaching the destination site. Specific stimuli induce local mRNA translation. Synaptic activation translates mRNAs localized in mature dendrites, whereas guidance cues stimulate translation in growing axons (reviewed in ref. ^1^). RBPs can also coordinate translational activation. For example, CPEB binds to cytoplasmic polyadenylation elements present in mRNAs, which can either repress or activate translation depending on its phosphorylation status^14^. Not surprisingly, numerous human pathologies and neurological disorders, such as Amyotrophic Lateral Sclerosis (ALS) and Fragile X syndrome (reviewed in ref. ^15^), are associated with mutations in RBPs and a failure to localize or translate certain mRNAs and proteins at specific subcellular compartments.

However, proteins become localized not only through (1) the localization and (2) local translation of the mRNAs encoding them but also (3) as a part of trafficking messenger ribonucleoprotein complexes or vesicles. Although current genome-wide studies have demonstrated the presence of thousands of mRNAs in neurites^4–8, 10, 11^, surprisingly, no systematic analysis has been carried out to assess the relationship and extent to which mRNA localization contributes to asymmetric protein localization in neurons. Indeed, previous studies have focused on the identification of the RNAs, largely leaving out analysis of the local proteome, or detecting the mere presence of mRNAs or proteins in neurites rather than relative enrichment.

Here, we sought to determine the extent by which each of these mechanisms contributes to the overall asymmetry of neuronal protein distribution and the importance of separate localization mechanisms. We perform RNA sequencing (RNA-seq), Ribo-seq, and mass spectrometry analyses on the neurites and soma of neurons differentiated from mouse embryonic stem cells (mESCs). We quantify 7,323 proteins and measure the levels and translation rates of the entire transcriptome. Using this approach, we identify hundreds of localized and locally translated transcripts, as well as localized proteins, and independently validate a number of candidates with important neuronal fuctions. Most remarkably, we find that almost half of the neurite-enriched proteome is encoded by neurite-localized mRNAs, revealing that mRNA localization is a key mechanism of protein localization to neurites. Moreover, as RBPs are key factors in RNA metabolism, we also identify 29 neurite-targeted RBPs, including both known components of the mRNA localization machinery and potential novel factors in mRNA transport and local translation. In addition, we identify dozens of neurite-targeted non-coding RNAs, including 12 long non-coding RNAs (lncRNAs) and 41 circular RNAs (circRNAs), with potential roles in neuronal polarity.

## Results

### Identification of the neurite-localized proteome

**Fig. 1:**
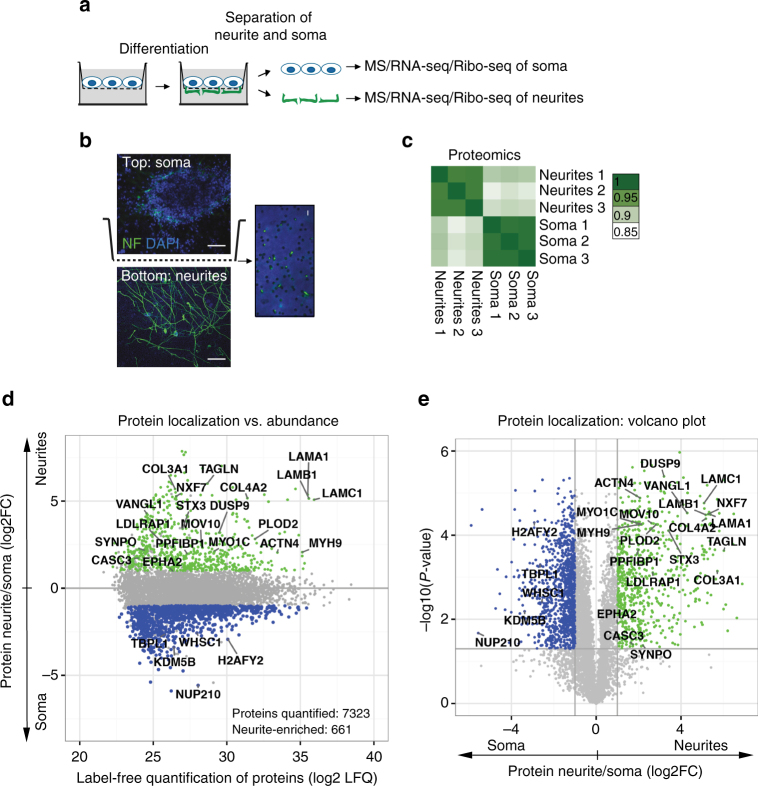
Local proteome of iNeurons. **a** Separation scheme. iNeurons are grown on a microporous membrane so that cell outgrowths extend on the lower coated side of the membrane to enable separation of the two cellular compartments (soma and neurites). **b** Fluorescent micrographs of the iNeurons differentiated on a microporous membrane described in **a**. Images taken above (*top*) and below (*bottom*) the membrane. Neurofilament immunostained neurites (*green*) extend on the lower side of the membrane, whereas soma (DAPI, *blue*) remain on the top, *scale bar* = 50 μm. The insert shows the magnification of the membrane with neurites growing through the membrane pores (*green*), *scale bar* = 5 μm. **c** Correlation heatmap of mass spectrometry replicates prepared from neurites and soma of iNeurons (three biological replicates in each case). Mass spectrometry samples were quantified using a label-free quantification method (LFQ). The numbers represent Pearson correlation coefficients of LFQ values. **d**, **e** Local proteome from neurites and soma. The data are presented as protein enrichment in neurites versus soma plotted against average LFQ intensities (*left*) and as a volcano plot (*right*). *Green*: neurite-localized proteins (log2FC > 1, *P*-values < 0.05); *blue*: soma-localized proteins (log2FC < −1, *P*-values < 0.05)

We sought to identify proteins and RNAs asymmetrically localized between the neurites and cell bodies (soma) in neurons, so we employed an assay that permits separation into distinct cellular compartments for spatial transcriptomic and proteomic analyses (Fig. 1). As a test system, we used neurons differentiated from mESCs by inducible expression of a pioneer proneural transcription factor ASCL1 (iNeurons for induced neurons). iNeurons represent a very well-characterized test system with all basic neuronal properties: they express mature neuronal markers, exhibit typical passive and active intrinsic membrane properties, and form functional pre- and postsynaptic structures^16, 17^. Moreover, due to overexpression of ASCL1 in every cell, they form highly homogenous population^18^ and can be generated in large amounts, which is critical for -omics approaches. We also confirmed the neuronal identity of iNeurons using the mass spectrometry-based approach SILAC (stable isotope labeling by amino acids in cell culture) to uninduced mESC and iNeurons. This method detects differences in protein abundance between samples using non-radioactive isotopic labeling^19^. Gene Ontology (GO) term overrepresentation analysis showed that proteins upregulated upon differentiation (iNeurons/mESC > 4) are associated with neuronal functions (Supplementary Fig. 1 and Supplementary Data 1). Finally, iNeurons expressed mature neuronal markers (Supplementary Fig. 2). Other means to obtain neurons (e.g., primary cortical neurons or traditional mESC differentiation systems that rely on exogenous differentiation factors added to the medium) do not produce large enough quantities of cultures that would be composed exclusively of neurons, let alone a particular class of neurons. Although immunopanning and fluorescence-activated cell sorting techniques have been successfully applied to purify populations of primary neurons genetically labeled with a fluorescent marker^20^, the procedure often adversely affects the viability of fragile cells such as neurons, limiting the amounts of recovered material. Although without the complexity of primary neurons, iNeurons represent a rapid and easily scalable system that allows initial discovery to then be validated in primary cells.

iNeurons were differentiated and maintained on a porous membrane support, such that soma stayed on the upper side of the membrane. The coating agent on the lower side of the membrane provided cues to stimulate neurite growth through the pores on the lower side of the membrane^9, 10^ (Fig. 1a). Immunostaning and western blotting for nuclear and neurite markers demonstrated that neurites were efficiently separated from cell bodies by the membrane (Fig. 1b and Supplementary Fig. 3). Indeed, neurofilament-rich neurites were found primarily on the lower side of the membrane, whereas soma, visualized with 4′,6-diamidino-2-phenylindole (DAPI), were only present on the top on the membrane. We then manually isolated neurites and soma from either side of the membrane for proteomic and transcriptomic analyses.

We subjected isolated neurites and soma to liquid chromatography–tandem mass spectrometry (LC–MS/MS) to identify their local proteomes. We measured 7,323 proteins using a label-free quantification (LFQ) method^21^. The analysis of three biological replicates showed a high correlation of LFQ values within each sample, whereas the correlation between neurite and soma samples was lower as expected (Fig. 1c). For each protein, we estimated its relative enrichment in neurites as the fold change (FC) of protein abundance between neurites and soma fractions. Thus, proteins with log2FC > 0 are enriched in neurites, and proteins with log2FC < 0 are enriched in the soma. For proteins detected in only one compartment sample (neurites or soma), we substituted the missing value with imputed data (see Supplementary Methods). We identified 661 proteins enriched in neurites by more than 2-fold when compared with soma (*P*-values < 0.05; Figs. 1d, e, *green* and Supplementary Data 2). As expected, nuclear proteins, such as histones and nuclear pore components, were localized in the somatic compartment, whereas neurites were enriched with components for the cytoskeleton, vesicular trafficking, adhesion molecules, and other synaptic markers (Figs. 1d, e and Supplementary Data 3). Thus, the proteomics data further confirms the efficient enrichment of soma and neurites fractions.

### RNA localization determines protein localization to neurites

**Fig. 2:**
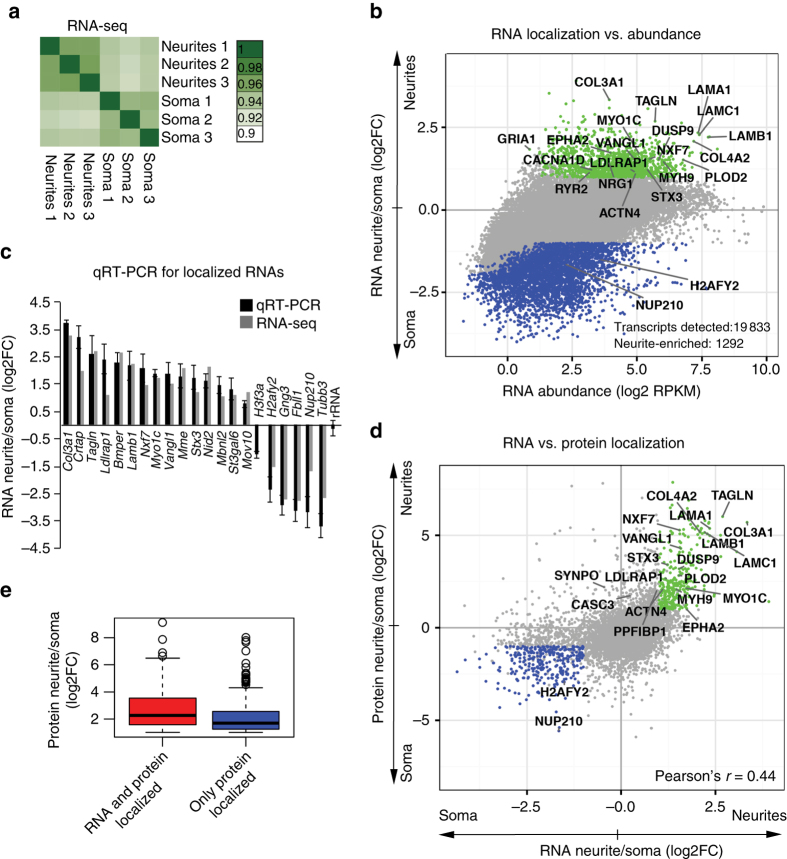
RNA localization determines protein localization to neurites. **a** Correlation heatmap of individual RNA-seq libraries prepared from neurites and soma of iNeurons (three biological replicates). Numbers indicate Pearson’s correlation coefficients. **b** Local transcriptome from neurites and soma. RNA-seq data are presented as RPKM (reads per kilobase of transcript per million mapped reads) and shown as in Fig. 1d. **c** qRT-PCR for selected neurite-localized RNAs identified by RNA-seq. Histone RNAs and rRNA used as soma-localized and unlocalized controls. Error bars represent SD. **d** RNA and protein enrichement in neurites of iNeurons. *Green*: neurite-localized proteins and RNAs (log2FC > 1, *P*-values < 0.05); *blue*: soma-localized proteins and RNAs (log2FC < −1, *P*-values < 0.05). **e** Average protein enrichment in neurites plotted for the group of genes localized at both protein and RNA level (*red*) and genes that are localized only at the protein level (*blue*; log2FC > 1, *P*-values < 0.05)

Three mechanisms contribute to protein localization within a cell: the transport of synthesized proteins, mRNA localization, and local translation. To identify proteins localized through mRNA localization, we sought to detect neurite-enriched mRNAs. We performed strand-specific total RNA-seq from the soma and neurites to quantify the local transcriptome. We observed a high correlation between three biological replicates of the RNA-seq libraries, which demonstrates reproducibility in our transcriptomic data (Fig. 2a and Supplementary Data 2; for mapping statistics, see Supplementary Data 4). We quantified 18,111 protein-coding transcripts in neurites and 19,833 in soma with a threshold > 1 RPKM (reads per kilobase of transcript per million mapped reads, Fig. 2b). RNA localization to neurites in our transcriptomic analysis was estimated as a FC of RNA abundance between neurites and soma. We identified 1,292 transcripts enriched in neurites by at least 2-fold when compared with the soma (*P*-values < 0.05).

We found transcripts known to be preferentially localized, such as syntaxin-3 (*Stx3*)^22^, glutamate receptor-1 (*Gria1*)^5^, calcium channel *Ryr2* ^5^, inositol 1,4,5-trisphosphate receptor type 1 (*Itpr1*)^5^, neuregulins (*Nrg1* ^23^ and *Nrg2* ^24^), voltage-dependent l-type calcium channel subunit α-1D (*Cacna1d*)^5^, ephrin type-A receptor 2 (*Epha2*)^25^, unconventional myosin-Ic (*Myo1c*)^26^, low-density lipoprotein receptor adapter protein 1 (*Ldlrap1*)^27^, vang-like protein (*Vangl*)^28^, and transcripts encoding mitochondrial proteins^6, 10^, consistent with previous works (see also Supplementary Data 5). We validated 21 localized RNAs with quantitative reverse transcriptase–PCR (Fig. 2c).

To determine the extent by which mRNA localization contributes to protein localization, we compared neurite enrichment at both the protein and mRNA level. Importantly, we observed a statistically significant correlation (Pearson’s correlation coefficient 0.44, *P*-value < 2.2 × 10^−16^) between protein and RNA localization to neurites (Fig. 2d). This result indicates that mRNA localization accounts for a substantial fraction of the neurite-localized proteome (303 out of 661 proteins; log2FC > 1, *P*-values < 0.05 for RNA-seq and proteomics). Interestingly, this fraction represents the proteins highly enriched in neurites (Fig. 2e), suggesting that the accumulation of high amounts of local protein requires mRNA localization.

### Neurite-targeted mRNAs are locally translated

**Fig. 3:**
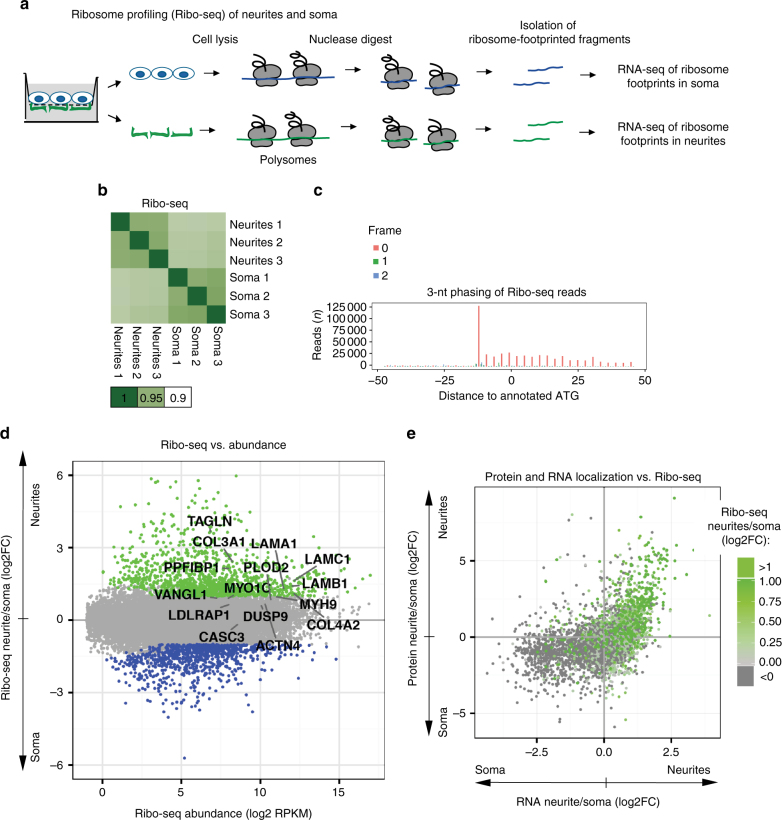
Ribo-seq of neurites and soma demonstrates that neurite-enriched proteins are locally translated. **a** Schematic representation of local Ribo-seq. **b** Correlation heatmap of individual Ribo-seq libraries, prepared from neurites and soma of iNeurons (three biological replicates). **c** Ribo-seq reads show subcodon resolution supported by a strong bias toward the translated frame (frame 0) and 3 nt periodicity. Read length: 29 nt. **d** Local Ribo-seq from neurites and soma. Enrichment of Ribo-seq reads in neurite versus soma plotted against average abundance of Ribo-seq reads (RPKM mapped to CDS). *Green*: transcripts preferentially translated in neurites (neurites/soma log2FC > 1, *P*-values < 0.05). **e** RNA enrichment in neurites plotted against protein enrichment as in Fig. 2d, and color-coded for enrichment of Ribo-seq reads in neurites. *Green*: genes preferentially translated in neurites according the Ribo-seq data

The correlation between RNA and protein localization to neurites suggests that neurite-targeted mRNAs are locally translated. Thus, we quantified local translation in the neurites and soma of iNeurons separately by applying ribosome profiling (Ribo-seq), a technique that generates a snapshot of ribosome footprints on translated RNAs^29^ (Fig. 3a). We optimized the Ribo-seq protocol to accommodate the relatively low amounts of material obtainable from neurites. We compared three different protocols: (a) the widely used protocol by Ingolia et al.^29^, (b) a simplified method by Reid et al.^30^, and (c) a protocol we developed based on the Ingolia method (Supplementary Fig. 4). Protocol (c) assumed that given a unique ribosome footprint size (~28–30 nt), we could isolate ribosome-footprinted fragments by electrophoresis-based size selection and skip the ribosome purification step. This would allow us to recover more material and therefore minimize the amount of input. Indeed, both protocols (a) and (c) showed optimal performance, as estimated by mapping statistics, read length distribution, and resolution (Supplementary Fig. 4). We selected protocol (c) to generate translation snapshots of isolated soma and neurites.

We observed a high correlation between the three biological replicates from the Ribo-seq libraries (Fig. 3b). The mapping statistics of Ribo-seq reads are shown in Supplementary Fig. 5A. Most reads mapped within coding sequences, which reflects a fraction of translated mRNAs. Moreover, we observed subcodon resolution, the hallmark of translation. Subcodon resolution is a 3-nt periodic alignment pattern, which reflects the codon-by-codon movement of translating ribosomes along a transcript^31^ (see Fig. 3c for the cumulative plot and Supplementary Fig. 5B for individual replicates). We used the ratio of Ribo-seq reads in neurites versus soma to assess the relative translation amount in each cell compartment and refer to transcripts with at least twofold neurites/soma ratio as locally translated (Fig. 3d). Notably, comparison of Ribo-seq data with local transcriptome and proteome indicated preferential translation of localized RNAs and proteins in neurites (Fig. 3e, gradient of *green* for Ribo-seq neurites/soma FC; see also Supplementary Fig. 5C, D).

Next, we used pulsed SILAC (pSILAC) to evaluate local translation in the cellular compartments. pSILAC^32^ is a variation of SILAC where labeled amino acids are added to the growth medium for a short time to monitor differences in de novo protein synthesis. We incubated neurons grown on porous membranes with either heavy (H) or medium (M) isotope-labeled amino acids for 2 h and then separated cells into neurites and soma. We chose a relatively short labeling pulse to minimize any possible contribution of protein transport between the two compartments. We pooled differentially labeled neurites and soma lysates together for further proteomic analysis (H neurites + M soma in forward (fw) and M neurites + H soma in reverse (rev) experiment, Supplementary Fig. 6A). The fw and rev experiments represent “label swap” replicates to eliminate biases introduced by the labeling procedure. The ratios of peak intensities, H / M in fw experiment and M / H in rev experiment, quantify relative translation rates in neurites versus soma. Using this approach, we measured the translation rates of 242 proteins in the two compartments (Supplementary Data 2, the relatively low coverage is expected after a short 2-h labeling pulse). Importantly, we observed a strong correlation between relative translation rates measured by Ribo-seq and pSILAC (Supplementary Fig. 6B).

**Fig. 4:**
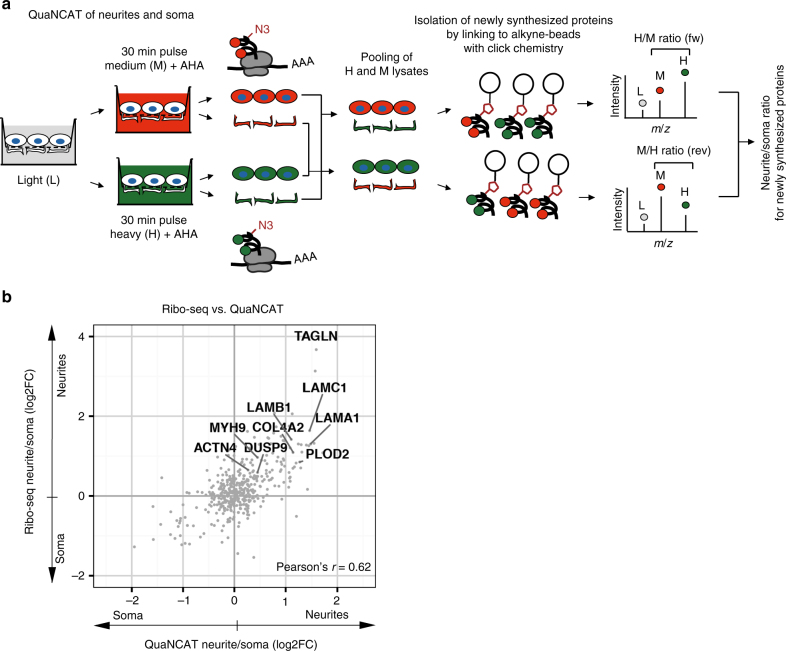
QuaNCAT of neurites and soma indicates local translation. **a** Scheme illustrating QuaNCAT of neurites and soma. H / M (forward experiment) and M / H (reverse experiment) ratios for each protein are the measures of neurite/soma translation rates. **b** Local translation rates measured by Ribo-seq correlate with QuaNCAT measurements. Averaged neurite/soma QuaNCAT ratios plotted against enrichment of Ribo-seq reads in neurites

Moreover, we applied QuaNCAT^33^ to quantify relative translation rates in neurites and soma. QuaNCAT combines pSILAC and labeling of nascent peptides with methionine analog azidohomoalanine (AHA; Fig. 4a). Newly synthesized proteins with incorporated AHA are enriched by covalently linking them to alkyne bearing agarose beads using “click chemistry.” Proteins are digested “on bead” and quantified by pSILAC labels. The purification step employed in QuaNCAT substantially reduces the background of pre-existing proteins, which enabled us to reproducibly measure relative protein abundance of 380 newly synthesized proteins after a short 30 min pulse of AHA. Relative translation rates measured by QuaNCAT supported our local Ribo-seq data (Pearson’s correlation coefficient 0.62, Fig. 4b), suggesting that a substantial fraction of the neurite-enriched proteome is indeed synthesized locally.

**Fig. 5:**
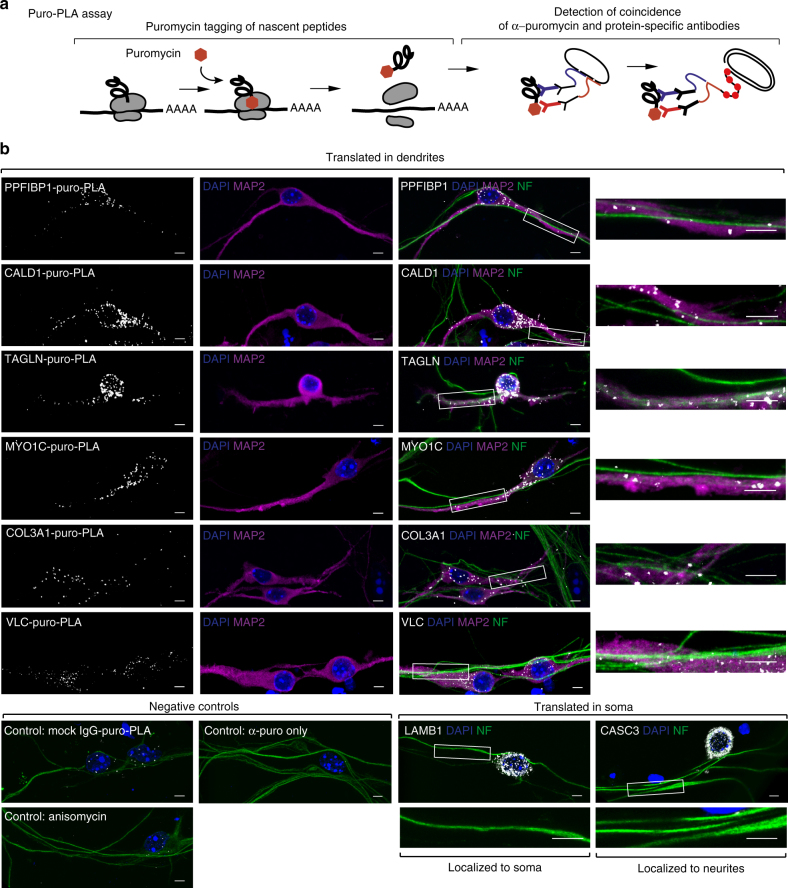
Validation of local translation by imaging. **a** Scheme illustrating the principle of puro-PLA assay to visualize specific newly synthesize proteins^34^. **b** Puro-PLA images of selected newly synthesized proteins in iNeurons. LMNB1 was used as reference for somatically produced proteins^34^. For negative control, cells were pretreated with the protein synthesis inhibitor anisomycin (anisomycin), protein-specific antibody was omitted (α-puro only) or mock rabbit IgG was used instead of specific antibody (mock IgG-puro-PLA). Immunostaining with MAP2 (*magenta*) and NF (*green*) enables detection of dendrites (MAP2-positive neurites) and axons (NF-positive, but MAP2-negative neurites). COL3A1, MYO1C, CALD1^67^ (Caldesmon), VCL^68^ (Vinculin), TAGLN^69^ (Transgelin), and PPFIBP1 are examples of neurite-translated proteins (Ribo-seq log2 neurites/soma = 2.4, 0.9, 1.2, 1.4, 3.7, and 2.1 correspondingly). Btz/CASC3 is a protein that showed no preferential translation in neurites (Ribo-seq log2 neurites/soma = −0.2). Magnifications of neurite sections (inserts) shown next to the images. *Scale bar* = 5 μm. Puro-PLA signal (*white*), NF (*green*), MAP2 (*magenta*), DAPI (*blue*). See also Supplementary Fig. 7 for puro-PLA validation on hippocampal neurons and different length of puromycin treatment

To visualize de novo synthesis of selected proteins in situ, we used the puro-PLA immunostaining assay (Fig. 5a), which uses puromycin-tagging of newly synthesized proteins^34^. Puromycin is a structural analog of the aminoacylated 3′-end of transfer RNA, which is incorporated into the nascent polypeptide chains, resulting in puromycin fusion proteins. The puro-PLA assay combines puromycin-tagging with the proximity-ligation assay (PLA) to detect the spatial coincidence between two antibodies: (1) an anti-puromycin antibody that binds de novo-produced proteins tagged with puromycin and (2) an antibody against a specific protein of interest. The secondary antibodies used in this assay are coupled to different oligonucleotide probes. Only when the two probes occur in close proximity can the linker oligonucleotide hybridize to both for rolling circle amplification. The amplified sequences are then detected by in situ hybridization.

Using this approach, we visualized the translation of three types of proteins: those translated in soma, those translated in neurites, and those translated in soma and subsequently localized to neurites. LMNB1 (Lamin-b1), a component of the nuclear lamina, served as a control for somatically translated proteins^34^ (Fig. 5b). We confirmed signal specificity with two types of negative controls: (1) pre-treating cells with anisomycin, a translation inhibitor that interferes with the peptidyl transferase reaction on the ribosome^35^ to inhibit puromycin incorporation into newly synthesized proteins (Fig. 5b, anisomycin) and (2) by omitting one of the primary antibodies or substituting them with mock IgG (Fig. 5b α-puro only and mock-IgG-puro-PLA). Both controls did show a substantial reduction in the signal.

After validating puro-PLA assay specificity, we visualized selected newly synthesized proteins. *Col3a1* encodes collagen III, whose loss leads to neocortical dyslamination in the mouse^36^. *Col3a1* represents an example of mRNA localized to neurites and locally translated, which causes an accumulation of the protein in neurites. Indeed, we observed a signal for newly synthesized COL3A1 in neurites. Other examples of neurite-translated proteins validated by puro-PLA include the motor protein MYO1C, implicated in the extension of neuronal growth cones^37^, PPFIBP1 (Liprin β-1), which has a role in synapse formation^38^, and several proteins involved in cytoskeleton organization and neurite elongation (Fig. 5b). Moreover, these proteins are also locally translated in neurites of mouse hippocampal neurons, indicating broad utility of our data (Supplementary Figs. 7 and 8). Barentsz (Btz)/CASC3 is a component of RNA localization machinery^39–41^ and we detected this protein enriched in neurites (Fig. 1d). However, our RNA-seq and Ribo-seq results indicate that its mRNA was neither localized nor preferentially locally translated in neurites. These results suggest that Btz/CASC3 is synthesized in the soma and localized to neurites as protein, probably in a complex with its target mRNAs. Indeed, using the puro-PLA assay, we detected newly synthesized Btz mainly in the soma. These results validate our -omics data for local translation through imaging approaches.

### Identification of neurite-localized circular and lncRNAs

Non-coding RNAs comprise a heterogenous and important group of genes with various roles in gene expression. Forty percent of lncRNAs show brain-specific expression patterns (reviewed in ref. ^42^); thus, we analyzed lncRNA expression in the neurites and soma of iNeurons (Supplementary Fig. 9A and Supplementary Data 2). We detected 550 annotated lncRNAs ( > 10 RPKM). Although the majority were localized to soma, 12 lncRNAs of unknown function exhibited over 2-fold enrichment in neurites (*P*-values < 0.05). This result suggests they could contribute to neuronal polarity.

**Fig. 6:**
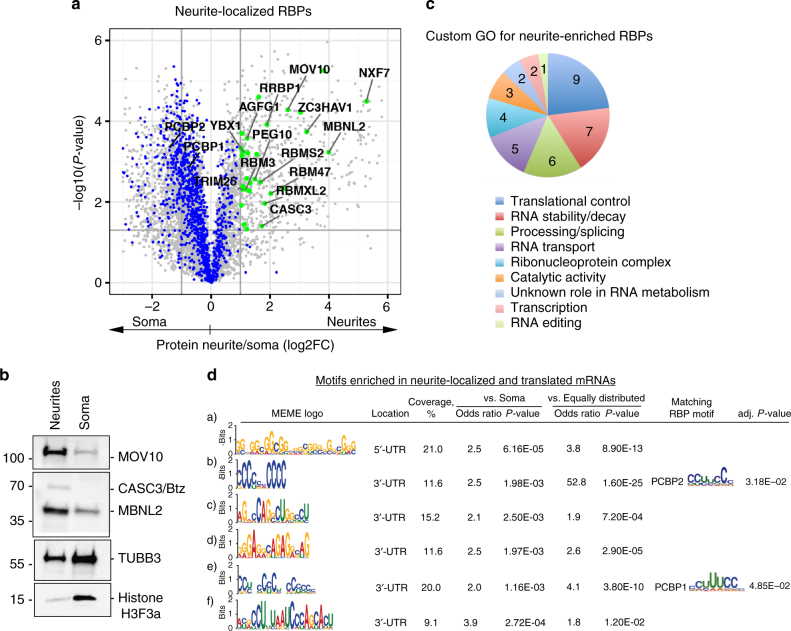
A subset of RBPs localizes to neurites. **a** Neurite-localized RBPs. Proteomic data from Fig. 1e were overlaid with available databases of mRNA-bound proteins^48–51^. Neurite-localized RBPs are highlighted in *green* (protein neurite/soma log2FC > 1, *P*-values < 0.05; see also Supplementary Data 2), the rest of RBPs are shown in *blue*. **b** Western blot validation for selected neurite-enriched RBPs. Histone H3 and TUBB3 were used as soma-enriched markers. **c** Manual annotation of neurite-enriched RBPs (protein neurite/soma log2FC > 1 and *P*-values < 0.05) for RNA-related functions. Number of proteins in a given category is indicated on the pie chart. Some RBPs were annotated to multiple GO categories. See also Supplementary Data 7. **d** Motifs found in mRNAs localized to neurites and locally translated. Motif discovery was done with MEME^56^ and enrichment calculations with MAST^57^. Fisher’s exact test was used to assess statistical significance of the association and its enrichment (odds ratio). Alignment of known RBP target sites^70^ (not restricted to neurite-localized RBPs identified in Fig. 6a) was performed using Tomtom^60^; only best hits are shown

circRNAs represent an important class of regulatory non-coding RNAs, which result from so-called “head-to-tail splicing” and are abundant in the brain (reviewed in ref. ^43^), particularly in synaptosome^44^. Some circRNAs function by binding and sequestering microRNAs^45, 46^. Neurite-localized circRNAs may participate in local RNA regulation by sequestering RBPs from RNAs. Interestingly, we found 90 genes for which neurite levels of circular transcripts were at least 2 times higher than levels of their linear counterparts (log2 Circ/Linear neurites > 1, Supplementary Fig. 9B and Supplementary Data 6). Of these, 41 show circRNA enrichment over linear transcripts only in neurites and not in soma (log2 Circ/Linear neurites > 1 and log2 Circ/Linear soma < 0). For example, the circular transcript for the *Ephb2*, a receptor tyrosine kinase that functions in axon guidance^47^, is > 10 times more abundant than its linear form in neurites. In the soma, this ratio is reversed, such that the linear *Ephb2* is ~7 times more abundant than the circular transcript. Different affinities to localization machinery may mediate the differential localization of linear and circular transcripts. We also cannot exclude the possibility of local splicing, especially as we find a number of splicing factors enriched in neurites (Supplementary Data 7 and Fig. 6a).

### Identification of neurite-localized RBPs

RBPs localized to neurites represent a particularly intriguing group, as they may bind a subset of neurite-localized mRNAs and regulate their localization, stability, or translation. Recent studies identified mRNA-bound RBPs in HEK293, HeLa, mESC, and yeast using an mRNA interactome capture approach^48–50^ and created a census of 1,542 RBPs^51^. We compared this data set with our local neuronal proteome to find RBPs (Supplementary Data 2, column “RBP”). Using this analysis, we identified 29 RBPs enriched in neurites over 2-fold (*P*-values < 0.05, Fig. 6a). We validated this neurite enrichment for several RBPs by western blotting (Fig. 6b). Our list includes several RBPs types (Fig. 6c and Supplementary Data 7, see also Supplementary Fig. 10A for GO term enrichment analysis): (1) RBPs involved in mRNA localization (e.g., Btz/CASC3 and MBNL2), (2) RBPs that regulate mRNA stability, translation, or splicing with no known neurite-specific functions (e.g., MOV10^52–54^), and (3) RBPs with no classified function. Btz/CASC3^39–41^ and MBNL2^10, 55^ represent known components of RNA localization machinery. Btz is a core component of the exon-junction complex, which is loaded onto nuclear mRNA and regulates different aspects of the mRNA life cycle, including localization. MBNL2 participates in alternative splicing, polyadenylation, and mRNA localization in neurons, and its inhibition is linked with RNA-mediated disease myotonic dystrophy. RBPs from groups (2) and (3) most likely have functions in the localization, stability or translation of neurite-enriched mRNAs.

RBPs can target RNAs based on the presence of linear motifs or secondary structures in their untranslated regions (UTRs). To identify de novo motifs associated with differential localization and translation in neurites, we used MEME^56^ and MAST^57^. We performed motif searches on 3′- and 5′-UTRs of mRNAs localized to neurites and preferentially translated there (neurites/soma logFC > 1, *P*-values < 0.05 for RNA-seq and proteomics). We used transcripts enriched and translated in soma (RNA-seq and proteomics logFC < −1, *P*-values < 0.05) or equally distributed (−0.58 < RNA-seq and proteomics neurites/soma logFC < 0.58) as a reference. We observed a significant enrichment for several motifs (Fig. 6d). Curiously, motif (a) found in 21% of neurite-localized and translated mRNAs is reminiscent of GC-rich motifs associated with m^1^A methylation sites in mRNA 5′-UTRs, reported to promote translation^58^. Indeed, our analysis of the m^1^A and m^6^A sites, that have been experimentally validated in mouse liver, mouse embryonic fibroblasts, mESCs, and brain^58, 59^, showed a significant enrichment of m^1^A sites among mRNAs localized to neurites and translated there (Supplementary Fig. 10B).

Some de novo identified motifs match known RBP motifs revealed with the Tomtom program^60^, such as the hnRNP E/poly(rC)-binding proteins (PCBP) broadly involved in RNA metabolism^61^. PCBP2 regulates splicing of Mapt/Tau exon 10, which is critical for neuronal survival and function^62^. As alternative splicing has an important role in mRNA localization and translation^10, 11^, PCBPs may contribute to this process. These results suggest a link between the sequence elements in mRNAs and their localization and translation in neurites.

## Discussion

Proper subcellular localization of proteins is crucial for normal physiological function. It can be achieved (1) by transporting proteins with molecular motors as parts of RNPs or vesicular organelles, (2) through mRNA localization and local translation or (3) via preferential local translation of equally distributed mRNAs, i.e., due to localization-dependent translational regulation. Specific examples for each mechanism have been described in the literature, but it is unclear to what extent each contributes to the overall protein distribution asymmetry. One reason for this is that most genome-wide studies^2–8, 10–12^ focused on a particular level of gene expression (transcriptome, proteome, or translated transcriptome) or a single cellular compartment (e.g., axon without comparison with the soma). For example, Taliaferro et al.^10^ applied RNA-seq to neurites and the soma of neuronal cell lines and mouse cortical neurons, to study the differential localization of RNA isoforms. Shigeoka et al.^11^ used the Ribotag approach to identify ribosome-bound mRNAs in mouse retinal axons.

**Fig. 7:**
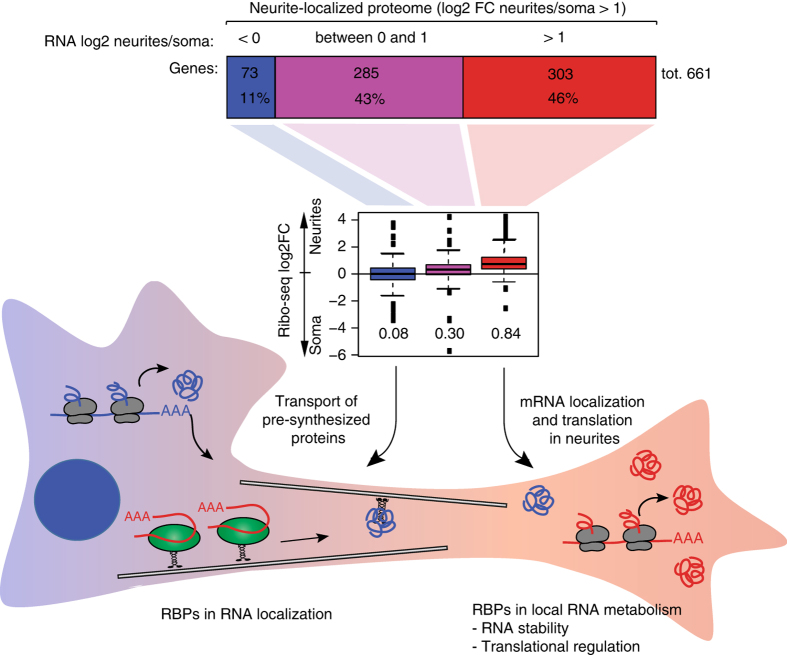
RNA localization as the key determinant of protein localization and potential functional roles for RBPs. Scheme illustrating the contribution of mRNA localization to neurite-localized proteome. Neurite-localized proteins (proteomics log2 neurites/soma > 1, *P*-values < 0.05) were split into three groups based on encoding mRNA localization pattern (*upper panel*): proteins encoded by localized mRNAs (RNA-seq log2 neurites/soma > 1, *P*-values < 0.05), proteins encoded by moderately enriched mRNA (0 < RNA-seq log2 neurites/soma < 1), and proteins localized to neurites without a significant contribution of mRNA localization (RNA-seq log2 neurites/soma < 0). Boxplot (*middle panel*) shows mean Ribo-seq log2 neurites/soma values for these three gene categories. Neuron schematic representation (*lower panel*) illustrates the predominant mechanism of protein localization through local translation of neurite-localized mRNAs

Here we used neuron fractionation scheme in combination with mass spectrometry, RNA-seq, Ribo-seq, and bioinformatic analyses to identify neuronal proteins and RNAs with distinct patterns of localization and translation in neurites and soma (Fig. 7). We specified a minimum twofold enrichment in one compartment over the other as the criteria for protein localization. Our analysis revealed that neurite-targeted mRNAs encode approximately half of the neurite-localized proteome (protein log2FC neurite/soma > 1, *P*-values < 0.05; 303 out of 661 proteins; RNA log2FC neurite/soma > 1, *P*-values < 0.05; see also Supplementary Data 8). Ribo-seq confirmed that this group of genes shows higher relative translation in neurites (Fig. 7, *middle panel*). Consistently with neurite localization, GO term enrichment analysis showed that these genes are associated with molecular functions “actin cytoskeleton”, “calcium ion binding”, “extracellular matrix,” and “growth factor binding” (Supplementary Data 8). Approximately 40% of these genes have neuron-related functions and are associated with neuronal diseases, including Alzheimer’s, Parkinson’s, and ALS (Supplementary Data 8).

A substantial group of the localized proteins are encoded by mRNAs, which are moderately enriched in neurites (285 out of 661 proteins; 0 < RNA log2FC < 1). These proteins may represent an intermediate group localized via multiple mechanisms, involving both mRNA and protein transport. A relatively small part of the local proteome cannot be explained by mRNA localization (73 out of 661 proteins; RNA log2FC < 0; protein log2FC > 1, *P*-values < 0.05). A protein transport mechanism may underlie half of these cases (34 out of 73 proteins; Ribo-seq log2FC < 0). Our data suggest that local translation without a significant contribution from mRNA localization mediates a small subfraction of the local proteome (10 out of 73 proteins; Ribo-seq log2FC > 1). The remainder could localize through a combination of both mechanisms (29 out of 73 proteins; 0 < Ribo-seq log2FC < 1). Interestingly, a recent study^9^ failed to detect a significant correlation between mRNA and protein localization when comparing protrusions and cell bodies in the MDA-MB231 breast cancer cell line, suggesting that mRNA localization is more critical or easier to detect in highly polarized cells with long extensions, such as neurites.

As RBPs are pivotal for RNA metabolism, we specifically examined and identified neurite-targeted RBPs (Fig. 6). We propose that these identified neurite-targeted RBPs likely (1) mediate mRNA localization or (2) regulate translation and/or stability of neurite-localized mRNA (Fig. 7), as we identified MBNL2 and Btz/CAS3, which are implicated in the regulation of mRNA localization^10, 39–41, 55^. We also found RBPs with known roles in mRNA decay and translational regulation such as MOV10, ZC3HAV1, which could regulate the levels of neurite-targeted transcripts and their translation efficiencies. Functions of other RBPs are under investigation, using knockout studies in combination with genome-wide target identification using RNA immunoprecipitation (RNA-IP) or crosslinking and immunoprecipitation (CLIP) assays, to provide additional insight into the mechanisms by which those RBPs establish cell polarity and neuron function. mRNAs localized and translated in neurites also show enrichment for specific sequence elements, including known RBP motifs and RNA modification sites (Fig. 6d and Supplementary Fig. 10). Consistently, prior studies report specific motif enrichment in neurite-localized^10^ and axonally translated mRNA isoforms^11^.

Our analysis provides the first combionatorial genome-wide snapshot of a local transcriptome, proteome and translated transcriptome that underlies cell polarity. These combined datasets provide a unique resource to promote future hypothesis-driven research. Importantly, our approach and results identified a key role for mRNA localization to establish cell polarity in developing neurons.

## Methods

### iNeuron culture and separation of neurites and soma

We generated mESCs with the doxycycline-inducible expression of ASCL1 as previously described^63^. mESC were grown on gelatin-coated flasks in 80% 2i/20% mESC medium (see below for media recipes). For differentiation into iNeurons and separation of soma and neurites, mESCs were allowed to form embryoid bodies (EBs) by growing in suspension in AK differentiation medium. After 1 day, EB formed from 10^6^ mESCs were plated on a Millicell six-well cell culture insert (PISP30R48 3 μm, Millipore), bottom-coated with Matrigel (356237, Corning). Cells were grown in a monolayer differentiation medium supplemented with 3 μg ml^−1^ doxycycline. iNeurons formed within 2 days after induction of ASCL1 with doxycycline. After 6 days, one compartment was removed using cotton swabs and the membrane with the remaining compartment (soma or neurites) was used for either RNA extraction with TRIFast (peqGOLD) or protein extraction with 8 M UREA, 0.1 M Tris-HCl pH 7.5.

2i medium: 50% Advanced DMEM/F12 (12634028 Thermo), 50% neurobasal (21103049 Thermo), 1× N2 (17502048 Thermo), 1× B27 (17504044 Thermo), 1 mM l-Glutamine (25030024 Thermo), 0.1 mM β-mercaptoethanol (βME), 10^3^ U ml^−1^ leukemia inhibitory factor (LIF, ESG1107 Merk Millipore), 3 µM CHIR99021, and 1 µM PD03259901 (130-104-170 Milenyi Biotec).

mESC medium: Knockout DMEM (10829018 Thermo), 14% fetal bovine serum (10439016 Thermo), 0.1 mM βME, 1 mM l-Glutamine, 1× MEM non-essential amino acid (11140035 Thermo), 1× nucleosides (ES008D Merck Millipore), and 10^3^ U ml^−1^ LIF.

AK differentiation medium: 50% Advanced DMEM/F12, 50% neurobasal, 10% knockout serum replacement (10828028 Thermo), 1 mM l-Glutamine, and 0.1 mM βME.

Monolayer differentiation medium: Advanced DMEM/F12 supplemented with B27, N2, and 3 μg ml^−1^ doxycycline.

### RNA-seq

Five hundred nanograms of total RNA isolated from neurites (isolated from iNeurons grown on ~3 Millicell inserts) or soma ( ~1/3 Millicell insert) was supplemented with ERCC RNA spike-in mix (4456740 Ambion) and used for library preparation with the Truseq stranded total RNA library prep kit (RS-122-2201 Illumina) according to the manufacturer’s recommendation. Each library was prepared in triplicate and sequenced on an Illumina NextSeq 500 sequencer with single-end 150 bp reads.

### Ribo-seq

iNeurons, grown on a Millicell insert, were treated with cycloheximide (100 μg ml^−1^), separated on neurites and soma as described earlier and frozen in liquid nitrogen. Twenty-one inserts were pooled together for neurites isolation and three inserts for soma isolation. Ribo-seq libraries were generated as previously described^29^ with some modifications. Each sample was lysed in 1 ml of polysome buffer (20 mM Tris pH 7.4, 150 mM NaCl, 5 mM MgCl_2_, 1 mM dithiothreitol (DTT), 1% Triton X-100, 100 μg ml^−1^ cycloheximide, and 5 U ml^−1^ Turbo DNase) and digested with 70 U of RNase I for 40 min at room temperature. As our analysis revealed that the quality and composition of the libraries generated with and without monosome recovery was comparable (Supplementary Fig. 4), we omitted the ribosome isolation step. After RNAse digestion, RNA was isolated using Direct-zol RNA MiniPrep (Biozym) and 400 ng of the isolated footprinted RNA were depleted of rRNA using RiboZero Gold rRNA removal kit (Illumina). The sample was concentrated using RNA clean and concentrator-5 kit (Biozym) and phosphorylated with 10 U T4 polynucleotide kinase for 30 min at 37 °C. The RNA was separated on a 15% Urea PAAG, 27–30 nt RNA fragments were eluted from the gel and used for library generation with Truseq small RNA library prep kit (RS-200-0012 Illumina) according to the manufacturer’s instructions. Each Ribo-seq library was prepared in triplicate and sequenced on an Illumina NextSeq 500 sequencer with single-end 75 bp reads.

### Proteomics

LC–MS/MS analysis was performed with in-solution digested protein lysates (neurites or soma, 20 μg) on a Q Exactive plus mass spectrometer (Thermo Scientific) as previously described^64^. LFQ was done using MaxQuant Analysis Software^65^.

For pSILAC^32^ iNeurons, grown on the Millicell insert for 6 days, were pulse labeled for 2 h using SILAC-customized monolayer differentiation medium, supplemented with Arg + 10 Da, Lys + 8 Da (H pulse), or Arg + 6 Da, Lys + 4 Da (M pulse). Soma and neurite lysates were prepared as described earlier and pooled as shown in Supplementary Fig. 6A before LC–MS/MS (H neurites + M soma for fw experiment and M neurites + H soma for rev experiment). The fw and rev experiments represent “label swap” replicates to eliminate biases introduced by the labeling procedure. The average of H / M (fw) and M / H (rev) ratios for each protein served as a measurement of the relative amount of translation in neurites compared with soma.

For SILAC experiments^19^, mESCs were grown in light (L) or H (Arg + 10 Da, Lys + 8 Da) SILAC 80% 2i/20% mESC medium for seven passages to ensure complete proteome labeling (97.96%). Labeled mESCs were further differentiated into iNeurons in SILAC-customized differentiation media (L or H). iNeurons and mESC lysates were pooled (H iNeurons + L mESC for fw and L mESC + H iNeurons for rev experiment) and subjected to LC–MS/MS. The averages of H/L (fw) and L/H (rev) ratios were used to measure relative protein abundance in iNeurons versus mESC.

For QuaNCAT experiments^33^, iNeurons were pulse labeled for 30 min in QuanCAT-customized DMEM medium (P04-02511 PAN) supplemented with 25 µM l- AHA (C10102 Thermo) and either Arg + 10 Da, Lys + 8 Da (H), or Arg + 6 Da, Lys + 4 Da (M). Lysates of subcellular compartments were prepared as described earlier and 2 mg of neurites and soma lysates were pooled as shown in Fig. 4a. For enrichment of newly synthesized AHA-containing proteins, we combined the pooled lysates with alkyne agarose beads (Thermo) and performed click chemistry as previously described^66^. In brief, the click reaction was performed overnight using the Click-iT protein enrichment kit (C10416 Thermo) according to the manufacturer’s instructions. Proteins were then denatured by adding DTT at 65 °C and alkylated by iodoacetamide, both “on bead.” The beads were then stringently sequentially washed in the following: (1) 1% SDS, 100 mM Tris pH 8.0, 250 mM NaCl; (2) 8 M urea, 100 mM Tris pH 8.0; and (3) 80% acetonitrile. Proteins were digested by Lys-C for 3 h and then by trypsin overnight. Newly synthesized proteins were identified by their incorporation of H and M SILAC amino acids. “Label swap” experiments, e.g., fw (H neurites + M soma) and rev (M neurites + H soma), were perfomed to eliminate biases introduced by the labeling procedure. The difference in proteins synthesized in the soma and neurites were quantified by the ratios H/M (fw experiment) and M/H (rev experiment).

### Puro-PLA and immunostaining

For imaging experiments on iNeurons, EB were grown in AK differentiation medium for 2 days, then ASCL1 expression was induced by adding 3 µg ml^−1^ doxycycline for another 2 days. EB were trypsinized, dissociated into single cells and plated on poly-dl-lysine-coated slides in a monolayer differentiation medium. After 5 days, cells were used for immunostaining or puro-PLA assay.

For conventional immunostaining, cells were fixed with 4% paraformaldehyde for 10 min and permeabilized with 0.2 % Triton X-100 in phosphate-buffered saline (PBS) for 10 min. After blocking with 1:5 dilution of the western blocking reagent (11921673001 Sigma) in PBS for 30 min, cells were probed with respective primary antibodies (ON at 4 °C), washed with PBS-Tween 0.05%, and incubated with fluorophore-coupled secondary antibodies for 1 h. Cells were mounted with ProLong Gold with DAPI (Cell Signaling). The following primary antibodies were used in immunostaining: mouse α-MAP2 antibody 1:1,000 (M4403 Sigma), chicken α-MAP2 antibody 1:1,000 (NB300213 Novusbio), guinea pig α-MAP2 antibody 1:200 (188004 Synaptic Systems), chicken α-Neurofilament antibody 1:10,000 (822601 Biolegend), mouse α-Neurofilament SMI312 antibody 1:10,000 (837904 BioLegend), rabbit α-Homer 1:100 (160003 Synaptic System), rabbit α-NeuN 1:100 (ABN78 Millipore), rabbit α-GAP43 1:50 (sc-10786), rabbit α-Tuj1/TUBB3 1:200 (T2200 Sigma), and rabbit α-Synapsin 1:200 (AB1543 Millipore). The secondary antibodies were used in 1:1,000 dilution: Alexa Fluor 488 goat α-chicken, Alexa Fluor 568 donkey α-rabbit, Alexa Fluor 568 goat α-chicken, Alexa Fluor 488 donkey α-mouse, and α-guinea pig Alexa Fluor 647. Imaging was performed using a Leica TCS SP5 confocal microscope with a × 63 oil objective. Images of cells growing on a porous insert were acquired with a × 40 oil objective and a pinhole of 90 μm as *Z*-stacks with 1,024 × 1,024 pixels *xy* resolution through the entire thickness of the cells and insert.

Puro-PLA was performed largely as previously described^34^. In brief, cells were incubated with 1 mg ml^−1^ puromycin for 15 min before fixation, unless otherwise indicated. For a negative control, cells were pretreated with 100 μg ml^−1^ anisomycin for 30 min before addition of puromycin. After fixation, cells were immunostained with α-puromycin antibody and an antibody against the protein of interest using Duolink reagent (DUO92008 Sigma) according to the manufacturer’s recommendations. The following primary antibodies were used in Puro-PLA: mouse α-puromycin 1:3,000 (3RH11 Kerafast) with one the rabbit IgGs: α-LMNB1 1:100 (sc-20682), α-COL3A1 1:50 (sc-8780-R), α-MYO1C 1:50 (EAP2048), α-PPFIBP1 1:50 (13961-1-AP), α-TAGLN 1:200 (ab155272), α-VCL 1:50 (ab129002), α-CALD1 1:25 (A304-163A Bethyl), and α-Btz/CASC3 1:50 (sc-98359). After Puro-PLA, cells were immunostained with chicken α-Neurofilament antibody 1:10,000 (822601 Biolegend) and guinea pig α-MAP2 antibody 1:200 (188004 Synaptic Systems) for 1 h in Duolink antibody diluent (DUO92002 Sigma), washed 3 × 10 min with PBS-Tween 0.05%, and incubated with α-chicken Alexa Fluor 488 and α-guinea pig Alexa Fluor 647 secondary antibodies for 1 h. Cells were mounted in Duolink in situ Mounting Medium. Images were acquired with a Leica TCS SP5 confocal microscope using × 63 oil objective and a pinhole setting of 60 μm. Images were processed with ImageJ (NIH).

### Western blotting

Enrichment of selected proteins in neurites and soma of iNeurons was validated by western blotting with the following primary antibodies: α-MOV10 1:5,000 (PLA0195 Sigma), α-Btz/CASC3 1:1,000 (sc-98359), α-MBNL2 1:5,000 (sc-136167), α-TUBB3 1:2,000 (T2200 Sigma), α-Histone H3 1:10,000 (ab1791 Abcam), and α-Neurofilament SMI312 antibody 1:10,000 (837904 BioLegend). Western blot images shown in Fig. 6b and Supplementary Fig. 3B have been cropped for presentation. Full-size images are presented in Supplementary Fig. 11.

### qRT–PCR

RNA from soma and neurites was treated with RQ1 DNase I, reverse-transcribed using the Maxima first strand complementary DNA synthesis kit (Thermo Fisher), and quantified by quantitative PCR (qPCR) using sensiFAST SYBR No ROX qPCR kit (Bioline). The following primers were used (PrimerBank ID): *Nxf7* (13561071a1), *Vangl1* (29164511a1), *ldlrap1* (160333774c1), *Col3a1* (20380522a1), *Crtap* (225543172c1), *Tagln* (291045204c1), *Bmper* (24371215c1), *Lamb1* (114326496c1), *Myo1c* (124494243c3), *Mme* (31543255a1), *Stx3* (924268a1), *Nid2* (26343027a1), *Mbnl2* (140971799c1), *St3gal6* (118130739c1), *Mov10* (254540178c1**)**, *H3f3a* (6680159a1), *H2afy2* (133892300c1), *Gng3* (6754022a1), *Fbll1* (148539881c1), *Nup210* (172073151c1), *Tubb3* (12963615a1), rRNA (fw: 5′-aaacggctaccacatccaag-3′, rev: 5′-cctccaatggatcctcgtta-3′). Relative neurites/soma expression levels were calculated using ΔΔ*C*_t_ method, with rRNA as a reference RNA.

### Data availability

The Next-Generation Sequencing (NGS) data reported in this paper are deposited on Array Express with the accession numbers E-MTAB-4978 (RNA-seq) and E-MTAB-4979 (Ribo-seq). Mass spectrometry data are deposited on ProteomeXchange with the identifiers PXD004640 and PXD005059.

## Electronic supplementary material

Supplementary Information

Supplementary Data 1

Supplementary Data 2

Supplementary Data 3

Supplementary Data 4

Supplementary Data 5

Supplementary Data 6

Supplementary Data 7

Supplementary Data 8
