# Supplementary material for: RNA localization is a key determinant of neurite-enriched proteome
Source: Nat Commun. 2017 Sep 19;8:583. doi: 10.1038/s41467-017-00690-6 (PMC5605627; doi:10.1038/s41467-017-00690-6)
Supplement: Supplementary file 1 — Supplementary Information [file 41467_2017_690_MOESM1_ESM.pdf]

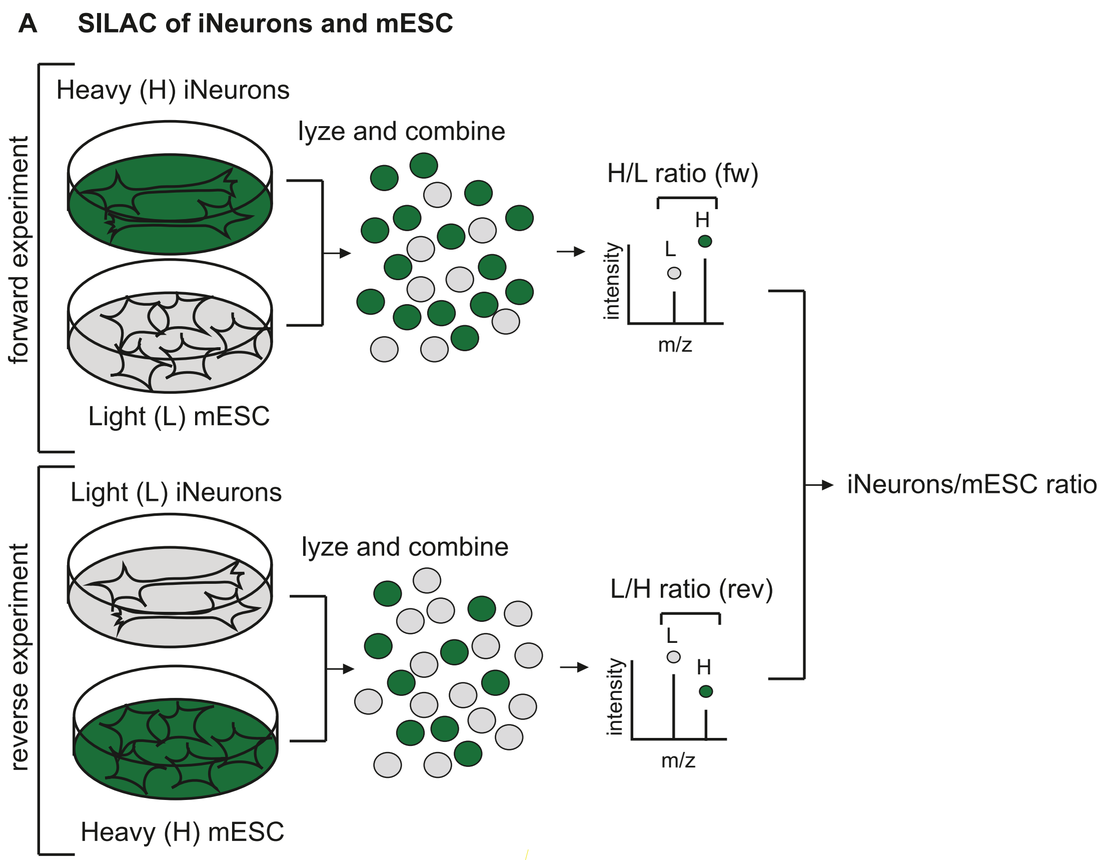

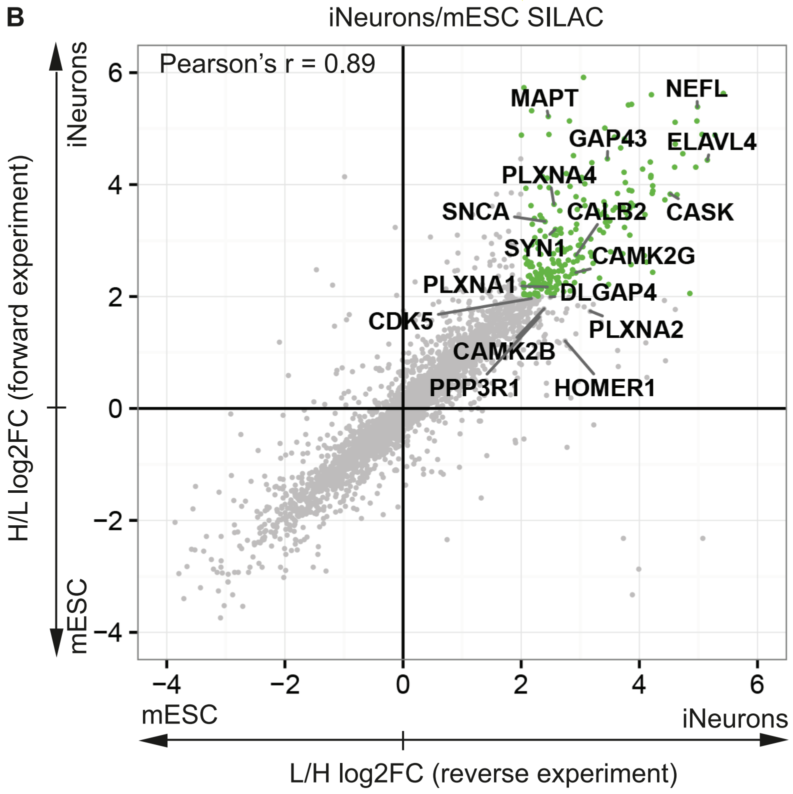

C

GO terms enriched in iNeurons:

calcineurin complex (GO:0005955)
semaphorin receptor complex (GO:0002116)
filopodium (GO:0030175)
myelin sheath (GO:0043209)
postsynaptic density (GO:0097481)
terminal bouton (GO:0043195)
site of polarized growth (GO:0030427)
growth cone (GO:0030426)
actomyosin (GO:0042641)
lamellipodium (GO:0030027)
actin filament (GO:0005884)
axon part (GO:0033267)
postsynaptic specialization (GO:0099572)
axon terminus (GO:0043679)
exocytic vesicle (GO:0070382)
neuron projection terminus (GO:0044306)
excitatory synapse (GO:0060076)
axon (GO:0030424)
neuron spine (GO:0044309)
synaptic vesicle (GO:0008021)

Supplementary Figure 1. ASCL1-induced Neurons (iNeurons) overexpress genes associated with neuron-related GO terms. (A) Schematic representation of SILAC (Stable isotope labeling with amino acids in cell culture) of mESC and iNeurons. Cells were cultured in either light (L) or heavy (H) medium, lyzed and pooled as indicated in the scheme (H iNeurons + L mESC in forward experiment and L iNeurons + H mESC in reverse experiment), followed by mass spectrometry analysis. H/L (forward) and L/H (reverse) ratios for each protein represent fold of protein overexpression in iNeurons. (**B**) Scatterplot of iNeurons/mESC protein ratios obtained in forward and reverse experiments. *Green*: proteins overexpressed in iNeurons by more than 4-fold in both forward and reverse experiments. (**C**) Top 20 'cellular component' GO terms associated with genes expressed upon differentiation of mESC into iNeurons (SILAC iNeurons/mESC log2 FC>2). The analysis was done using PANTHER (protein annotation through evolutionary relationship) classification system[21]. For the complete list, see **Supplementary Data 1**.

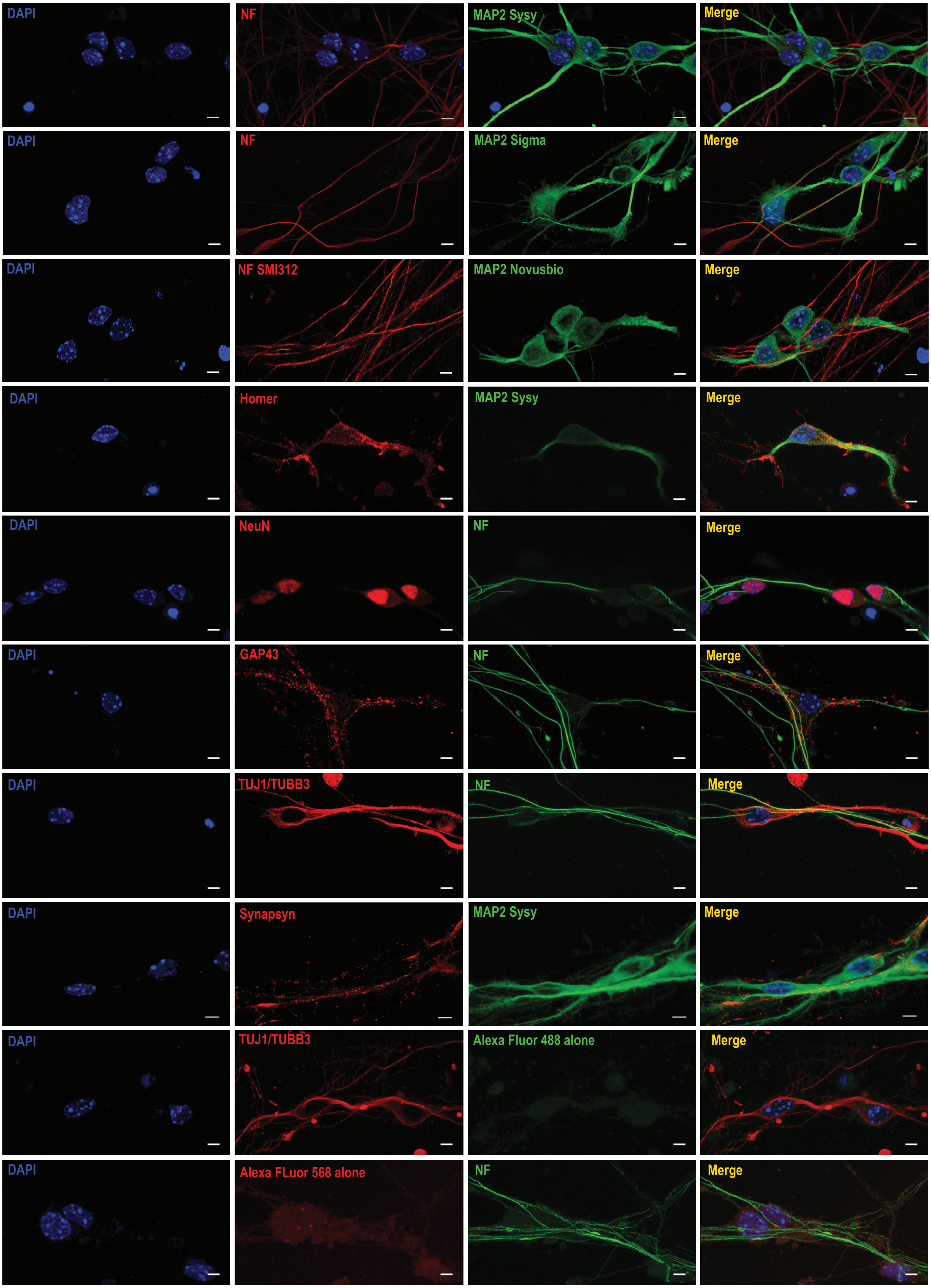

Supplementary Figure 2. iNeurons express mature neuronal markers. Fluorescent micrographs of the iNeurons immunostained with indicated neuronal (TUJ1/TUBB3, NeuN/RBFOX3, GAP43/Neuromodulin), dendritic (MAP2), presynaptic (synapsin), and postsynaptic (Homer) markers. Axons were distinguished by positive Neurofilament (NF) staining and absence of the MAP2 signal[32]. For negative controls, one of the primary antibodies was omitted (Alexa Fluor alone). Scale bar = 15 μm.

A

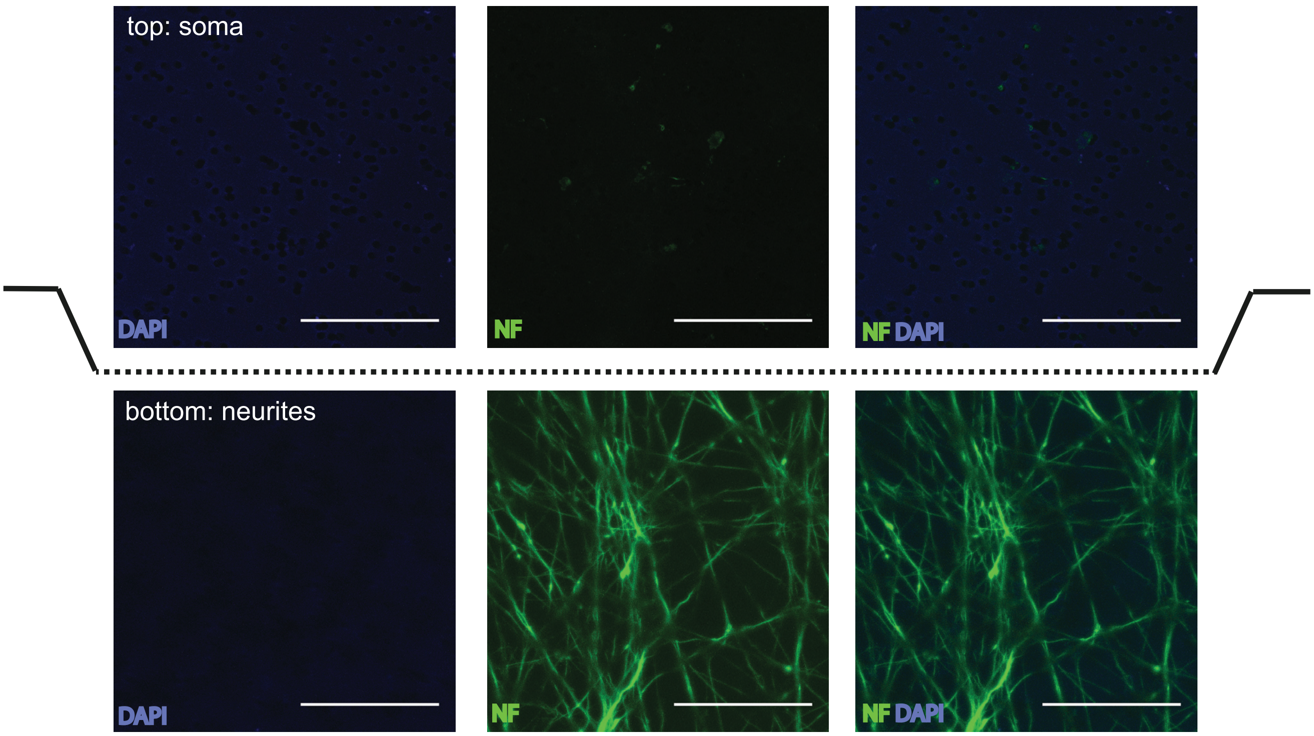

B

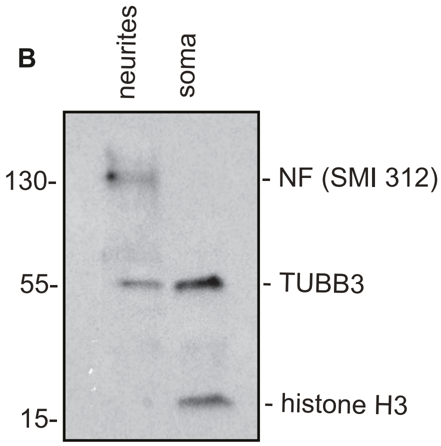

Supplementary Figure 3. Neurites and soma are efficiently separated with the microporous membrane. (**A**) Representative fluorescent micrographs of the neurites preparation, taken above (*top*) and below (*bottom*) the membrane. After differentiation on a microporous membrane, soma was removed from the top of the membrane as was done for all biochemical preparations of neurites. To estimate the efficiency of soma removal, the samples were stained with Neurofilament (*green*) for neurites and DAPI (*blue*) for soma, and the sample was examined for the presence of residual soma on the top of the membrane. Scale bar = 50 µm. (**B**) Protein lysates prepared from neurite and soma fractions were analyzed by western blotting. Histone H3 was used as a marker for soma and Neurofilament (NF) as a marker for neurites.

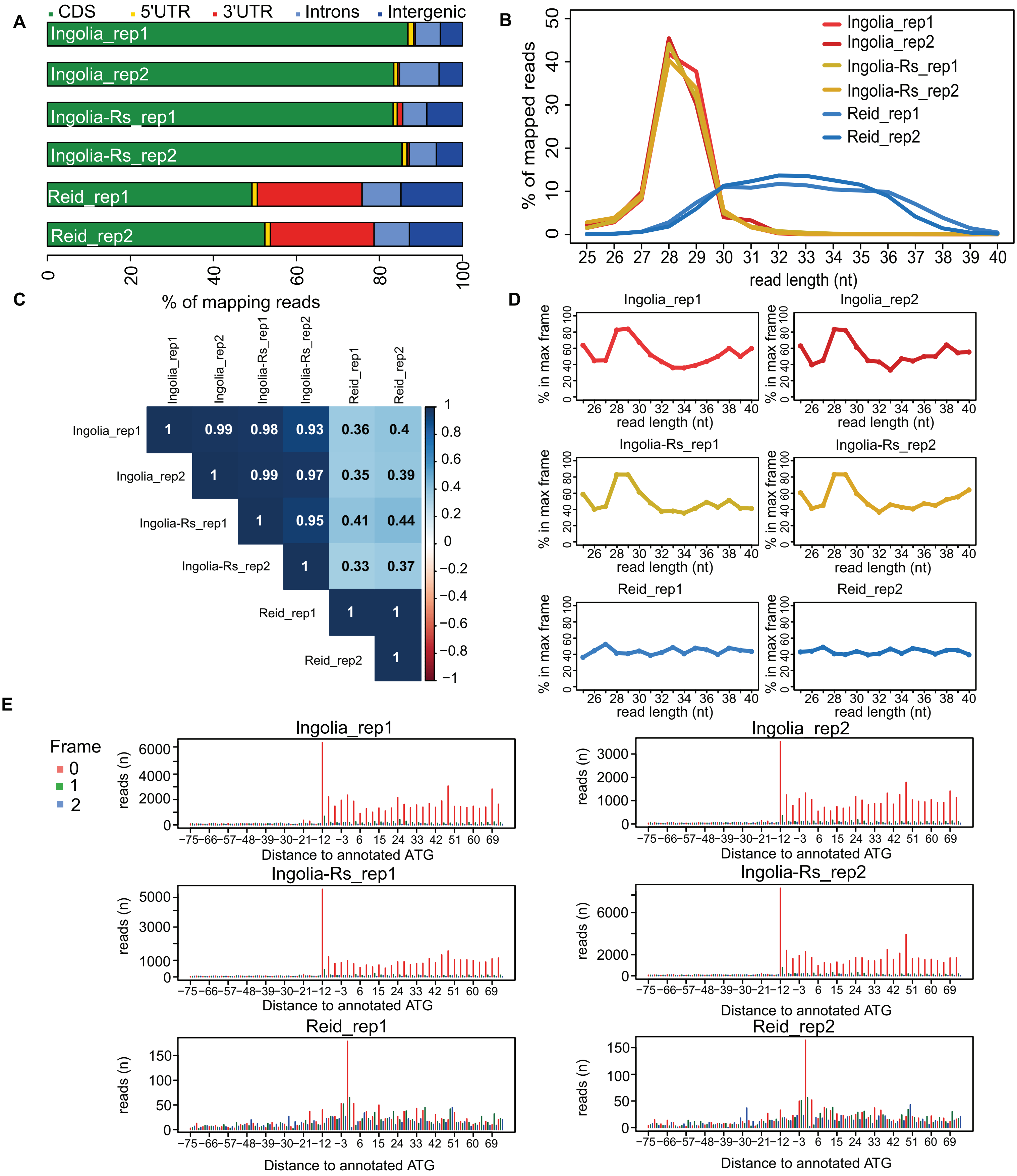

Supplementary Figure 4. Optimisation of the Ribo-seq protocol: the Ingolia's protocol with and without ribosome isolation results in a similar quality and composition of the libraries. Three different protocols were compared: Ingolia et al.[33], Ingolia's protocol with omitted ribosome isolation step (Ingolia-Rs), and a method of Reid et al.[34], each in two replicates (rep1 and rep2). The performance of each protocol was estimated based on read length and composition, percentage of in-frame reads, and 3-nt periodicity. (**A**) Barplot depicting mapping statistics of Ribo-seq reads. Most Ribo-seq reads obtained with Ingolia and Ingolia-Rs protocols map to CDS, reflecting the fraction of translated mRNAs. (**B**) Metagene plots showing length distribution of mapped Ribo-seq reads (**C**) Correlation heatmap of individual Ribo-seq libraries: high correlation is observed between the libraries generated with Ingolia and Ingolia-Rs, but not with Reid protocol. The numbers represent Pearson correlation coefficients (reads/gene in CDS). (**D**) Metagene plots showing the percentage of reads in frame relative to the read length: most reads obtained with Ingolia and Ingolia-Rs protocols are in frame with CDS. (**E**) Metagene aggregate plots displaying distance of 28-nt Ribo-seq reads from annotated start codon. Ribo-seq reads show subcodon resolution for Ingolia and Ingolia-Rs, but not for Reid protocol.

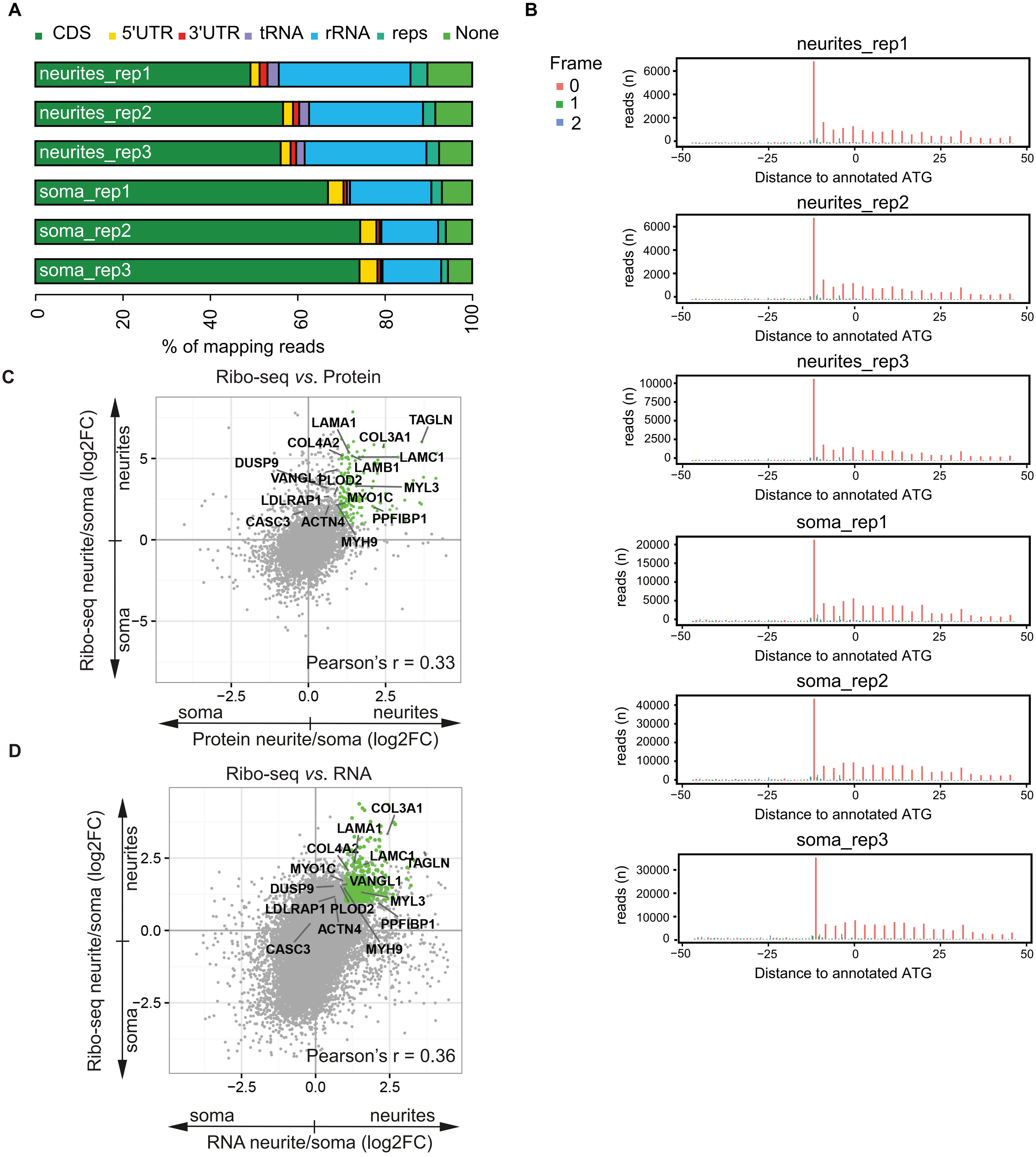

Supplementary Figure 5. Composition and quality of Ribo-seq libraries prepared from neurites and soma of iNeurons. (**A**) Barplot depicting mapping statistics of Ribo-seq reads in neurites and soma. Most Ribo-seq reads map to CDS, which reflect translated RNA fraction. (**B**) Plots displaying distance of Ribo-seq reads from annotated start codon for individual replicates of Ribo-seq libraries. All libraries show 3-nt periodicity of reads, reflecting codon-by-codon translation process. Read lenth: 29 nt. All plots were constructed using uniquely mapped reads. (**C**) RNAs localized to neurites are locally translated. RNA enrichment in neurites is plotted against enrichment of Ribo-seq reads in neurites. *Green*: RNAs that are enriched in neurites and locally translated (RNA-seq and Ribo-seq log2FC > 1, p-values < 0.05). (**D**) Proteins enriched in neurites are locally translated. Protein enrichment in neurites is plotted against enrichment of Ribo-seq reads in neurites. *Green*: Proteins that are enriched in neurites and locally translated there (log2FC > 1, p-values < 0.05).

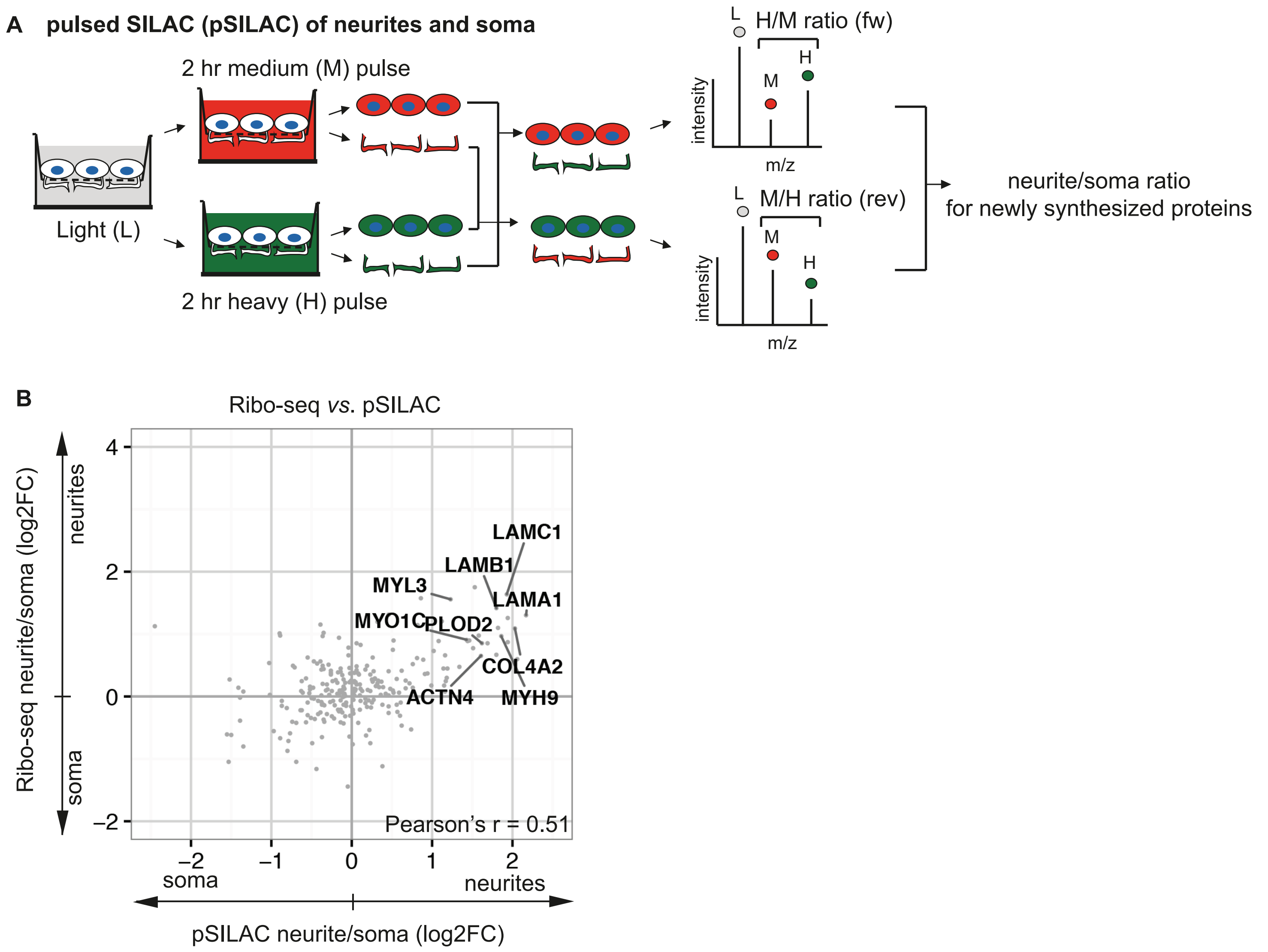

Supplementary Figure 6. Pulsed SILAC of neurites and soma indicates local translation. (**A**) Schematic representation of local pulsed SILAC (pSILAC). H/M (forward experiment) and M/H (reverse experiment) ratios for each protein are the measures of neurite/soma translation rates. (**B**) Local translation rates measured by Ribo-seq correlate with pSILAC measurements. Averaged neurite/soma pSILAC ratios are plotted against enrichment of Ribo-seq reads in neurites.

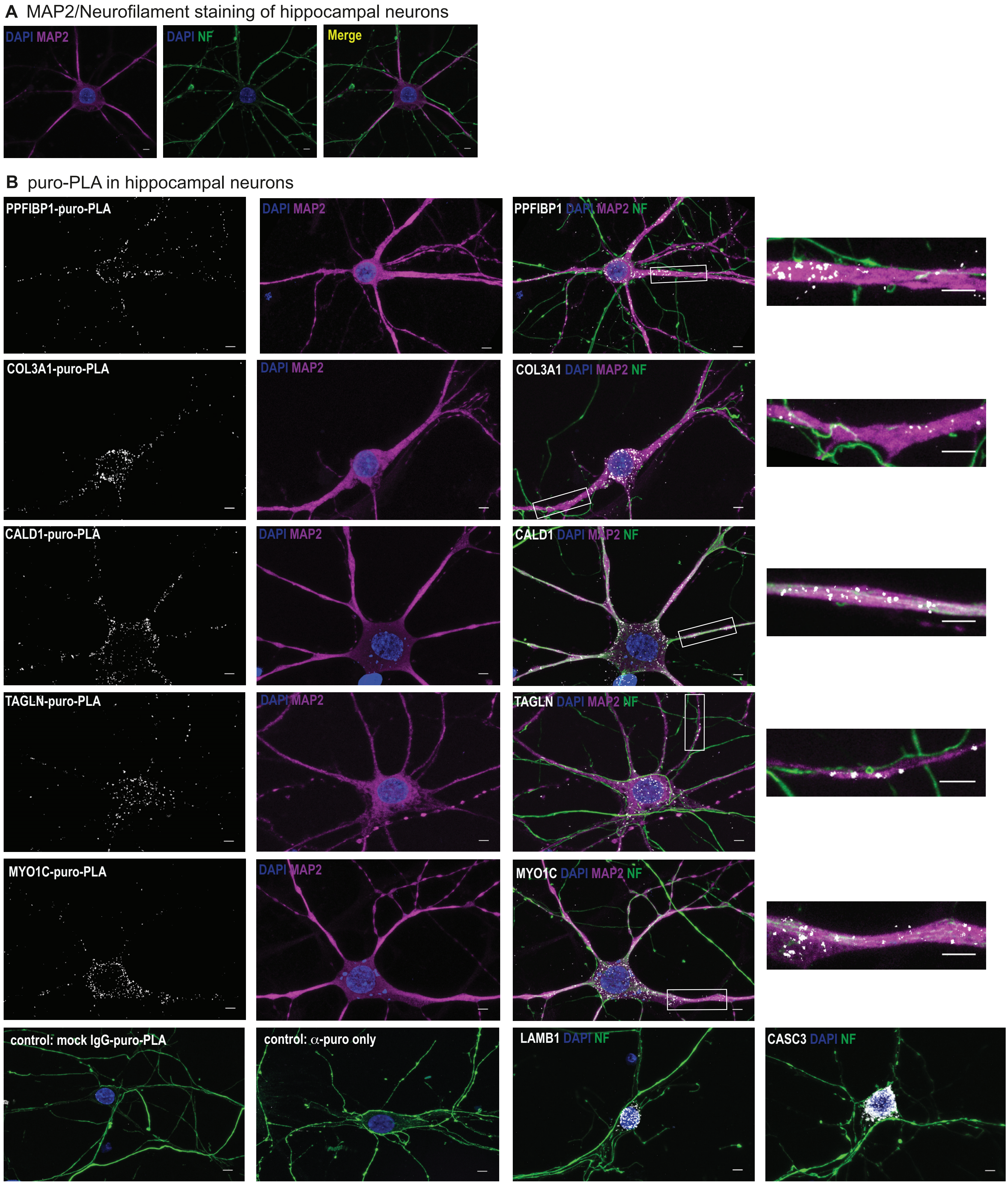

Supplementary Figure 7. Validation of local translation by imaging in mouse primary hippocampal neurons. (**A**) Immunostaining with MAP2 (*magenta*) and NF (*green*) enables detection of dendrites (MAP2-positive neurites[32]) and axons (NF-positive, but MAP2-negative neurites). (**B**) Puro-PLA images of selected newly synthesized proteins in primary hippocampal neurons. LMNB1 (Lamin-b1) was used as reference for somatically produced proteins[35]. For negative control, protein-specific antibody was omitted (*α-puro only*) or substituted with mock rabbit IgG (*mock IgG-puro-PLA)*. COL3A1, MYO1C, CALD1, TAGLN and PPFIBP1 are dendrite-translated proteins; and Btz/CASC3 is a somatically translated protein. Magnifications of neurite sections (inserts) shown next to the images. Shorter puromycin treatment (*5 min puromycin*) was implemented to ensure that neurite signal is due local synthesis rather than protein transport. Scale bar = 5μm. Puro-PLA signal (*white*), NF (*green*), MAP2 (*magenta*), DAPI (*blue*).

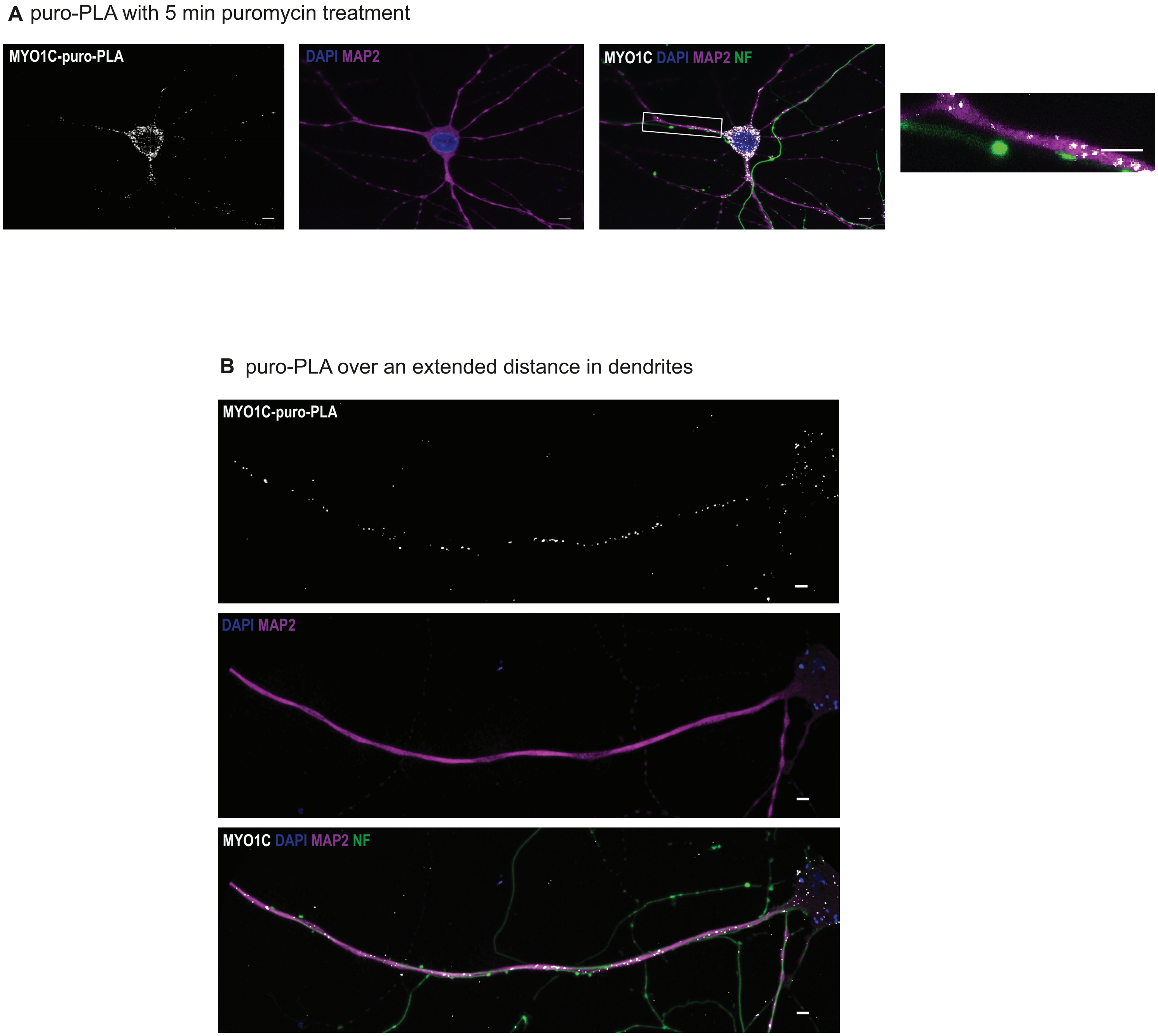

Supplementary Figure 8. Features of local translation in dendrites of primary hippocampal cultures. (**A**) Shorter puromycin treatment (5 min instead of 15 min) was implemented in MYO1C-puro-PLA (*white*) to ensure that neurite signal is due to local synthesis rather than protein transport. Scale bar = 5μm. DAPI (*blue*), Neurofilament (*green*), MAP2 (*magenta*). (**B**) The image of MYO1C-puro-PLA was taken over a larger field of view, to demonstrate that newly translated MYO1C can be detected at a long distance from soma. Scale bar = 5μm. MYO1C-puro-PLA (*white*), Neurofilament (*green*), MAP2 (*magenta*), DAPI (*blue*).

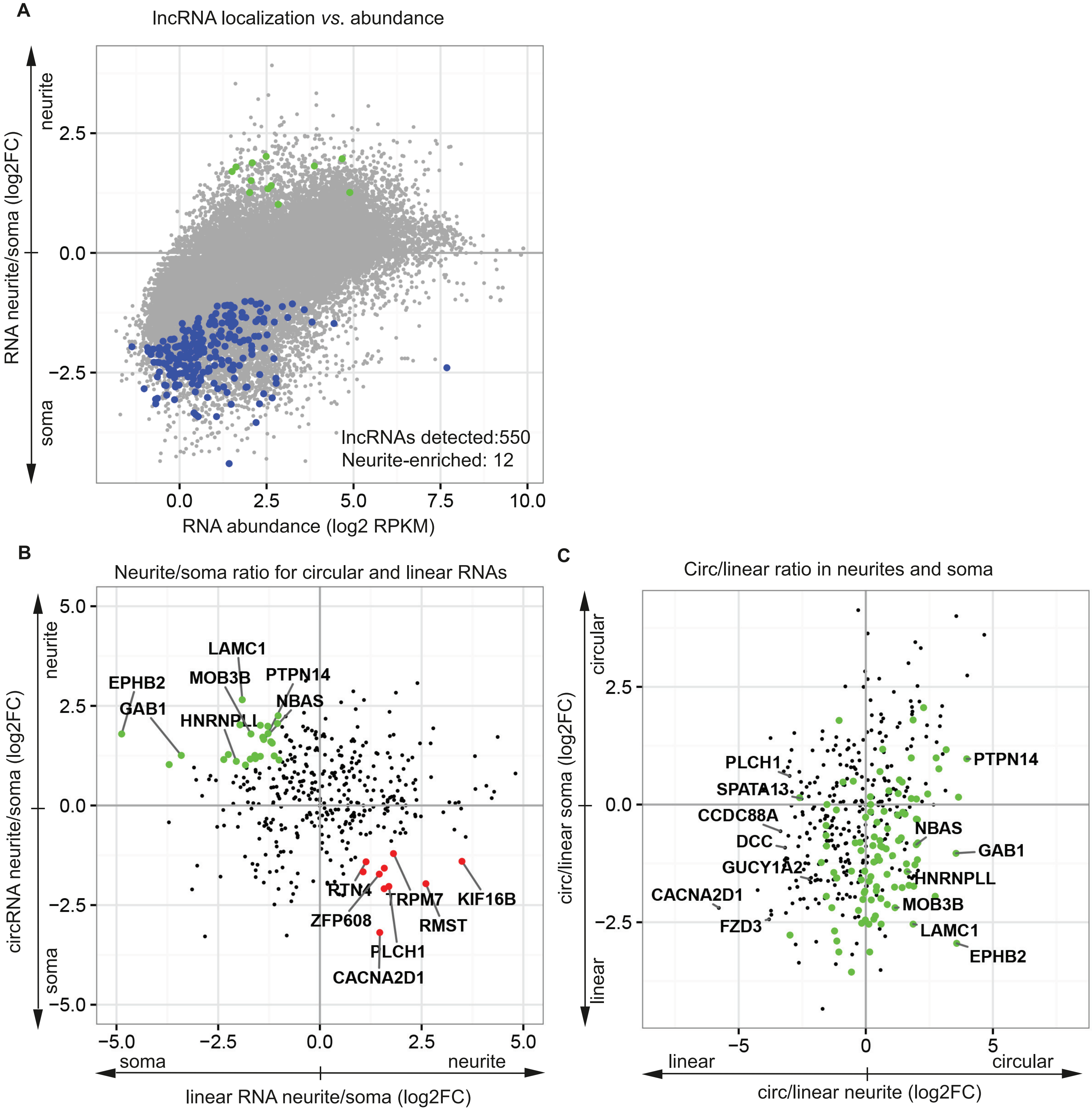

Supplementary Figure 9. Localized lncRNAs and circRNAs of iNeurons. (**A**) Neurite-localized lincRNAs. The data are presented as total RNA enrichment in neurites versus soma plotted against average RNA abundance. Green: neurite-localized lncRNAs (log2FC > 1, p-values < 0.05); blue: soma-localized lncRNAs (log2FC < -1, p-values < 0.05). (**B**) For a number of transcripts only one form - circular or linear - is localized to neurites. Enrichment of circRNAs in neurites was plotted against enrichment of linear RNAs in neurites. Green: transcripts that show enrichment of circular, but not linear forms in neurites (neurite/soma log2FC for circRNA > 1; for linear RNA < -1). Red: transcripts that show enrichment of linear, but not circular forms in neurites (neurite/soma log2FC for linear > 1; for circRNA < -1). (**C**) Neurites and soma show different ratios between circluar and linear RNA forms. Ratio between circular and linear form of transcripts in soma is plotted against the same ratio in neurites. Green: circRNAs that are at least 2-fold enriched in neurites compared with soma (circRNA log2FC neurites/soma > 1, p.values < 0.05).

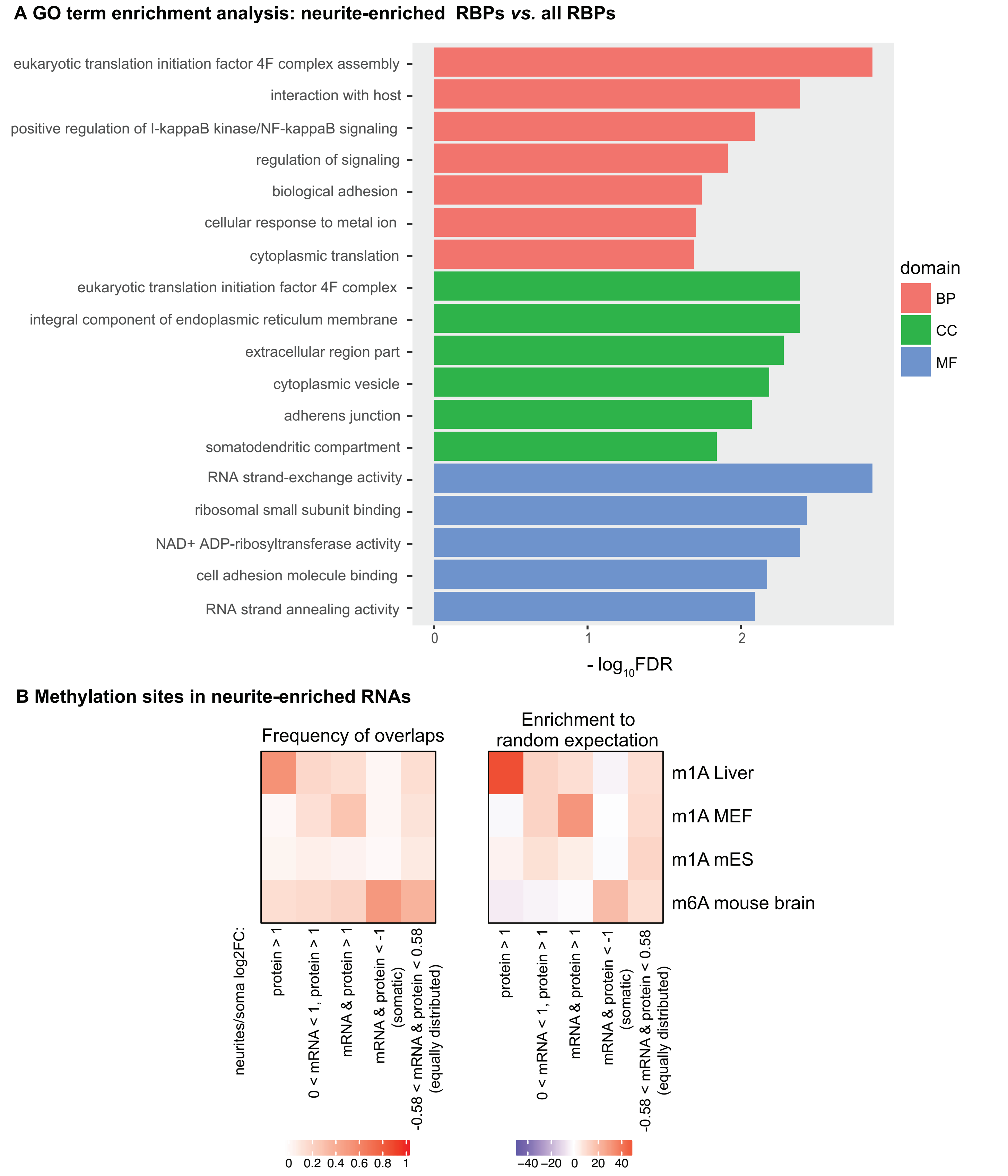

Supplementary Figure 10. Features of neurite-localized RBPs and transcripts. (**A**) GO term over-representation analysis for RBPs localized to neurites. gProfileR[22] was used to find enriched GO terms in neurite-localized RBPs (shown in Fig. 6A: enriched in neurites vs. soma by at least 2-fold, p-values < 0.05). As a background set we considered all the RBPs detected using mass spectrometry in our study. We only report GO terms with an enrichment FDR of less than 0.025. The three defined GO domains are shown: biological process (BP), cellular component (CC) and molecular function (MF). (**B**) Sequence features of neurite-localized transcripts. Experimentally determined m^{6}A and m^{1}A positions were overlapped with transcripts from each of the localization classes. Left heatmap shows the percentage of transcripts containing at least one post-transcriptionally modified position. The right heatmap shows the relative frequency measured in the number of standard deviations from the mean of the background distribution (gene classification and detailed calculation can be found in **Supplementary Methods**).

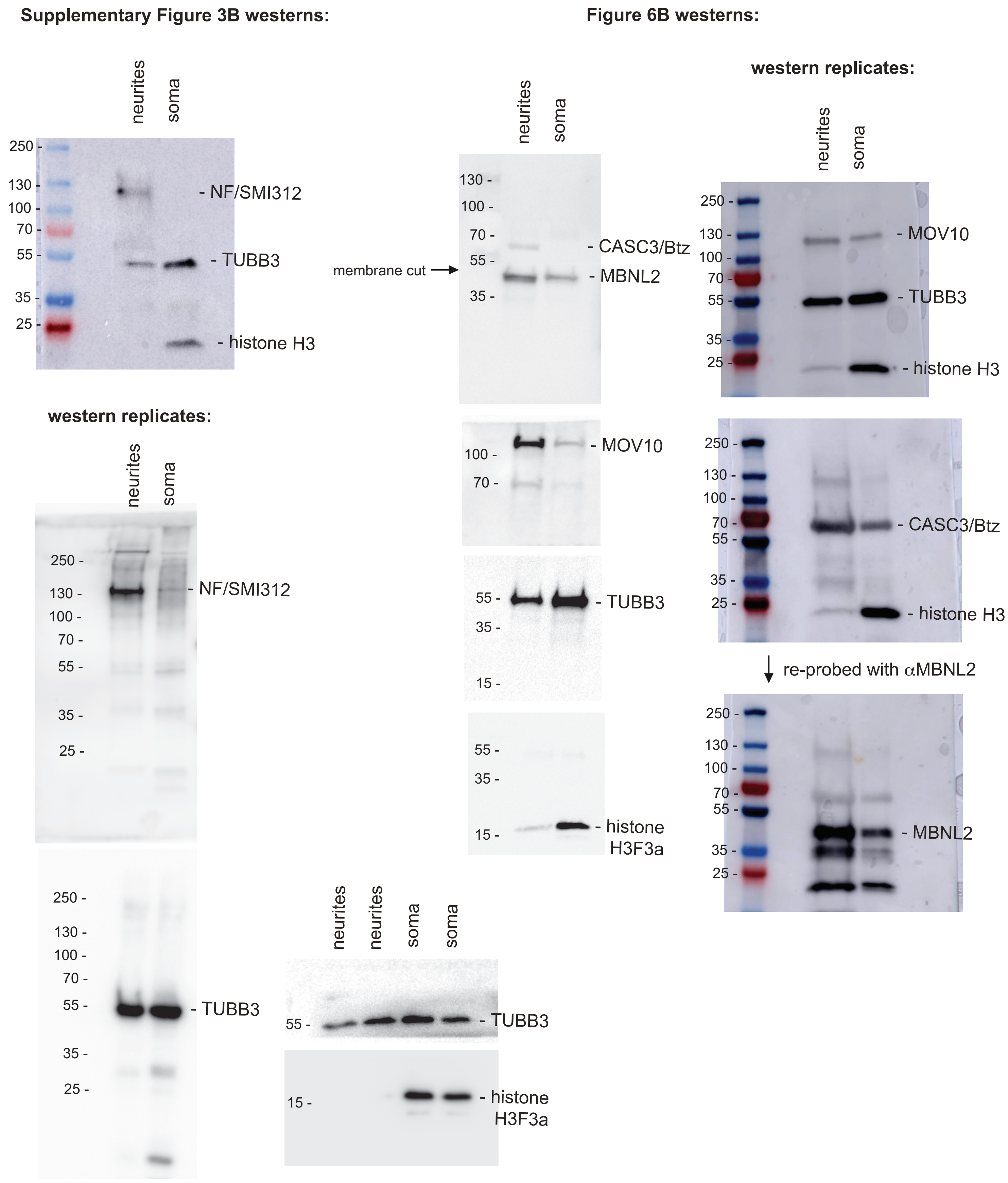

Supplemenary Figure 11. Full-size images of western blots shown in main Figure 6B and Supplementary Figure 3B. Because of the limiting amounts of material obtainable from neurites, each western blot mebrane was probed with multiple antibodies. For that, membranes were cut into parts corresponding to different ranges of protein molecular weights and incubated with the indicated antibody. In case of MBNL2 and CASC3/Btz probing, cut parts of membrane were re-joined before ECL and imaging. The replicate images with colorimetric markers were taken with a newer model Amersham Imager 600 that automatically generates an overlay of chemiluminescence signal with colorimetric markers.

Supplementary methods

Primary neuron cultures

For imaging experiments on iNeurons, EB were grown in AK differentiation medium for 2 days, then ASCL1 expression was induced by adding 3 μg/mL doxycycline for another 2 days. EB were trypsinized, dissociated into single cells and plated on poly-DL-Lysine-coated slides in a monolayer differentiation medium. After 5 days, cells were used for immunostaining or Puro-PLA assay.

Primary neuron cultures for immunocytochemistry were established according to the protocol of Kaech & Banker[1]. Briefly, hippocampi from newborn (P0) C57B/6 mice were dissected in cold GBSS (G9779 Sigma) supplemented with 100 U/ml penicillin and 0.1 mg/ml streptomycin solution. The tissue was transferred to a 1.5 ml Eppendorf tube containing 1ml of the Papain solution: 4.2 mg/ml papain (LS003126 Worthington) and 1mg/ml DNase I (Sigma, D5025) diluted in Enzyme solution (GBSS (G9779 Sigma), 0.2 mg/ml Cysteine, 1 mM $CaCl_2$, 0.5 mM EDTA, 3mM NaOH), 30 min at 37°C in a shaker. Afterwards, the papain solution was aspirated and the tissue was washed with 1ml/tube of Stop solution, which consisted of 1mg/ml DNase I (D5025 Sigma), 5% FBS (S0115 Biochrom), and 0.25% albumin. The tissue was slowly triturated using complete medium: MEM Eagle Modified (M2414 Sigma), 21 mM D-Glucose (1.08337.1000 Merck), 1x GlutaMax (K0302 Biochrom), 0.5x MEMVitamine (K0373 Biochrom), 1x Mito+ serum extender (355006 BD), 5% FBS (S0115 Biochrom). Dissociated cells were collected by centrifugation at 500g for 5 min, counted and plated at a density of 25.000 neurons per cm^2 on poly-L-lysine-coated coverslips[1] in DMEM/F12 GlutaMAX (31331028 Gibco), 1x B-27 (17504044 Thermo). Neurons were cultured for 4 weeks before imaging.

Mass spectrometry based proteomic analysis

An average yield of total protein obtained from one Milicell insert was ~30 μg for neurites isolation and ~375 μg for soma isolation. Proteins (neurites or soma, 20 μg) were lysed with 8 M urea, 0.1 M Tris-HCl pH 7.5 and digested by Lysyl endopeptidase (LysC) and trypsin. Digested peptides were desalted with C18 Stage Tips[2] prior to online liquid chromatography-tandem mass spectrometry (nanoLC-MS/MS) analysis. Peptides were separated on a monolithic column (100 μm i.d. x 2,000 mm, MonoCap C18 High Resolution 2000 [GL Sciences] at a flow rate of 300 nl/min with a 5 to 95 % acetonitrile gradient in 0.1% formic acid over 6 hours. QuaNCAT derived peptides were separated on a 15 cm column packed in house

with C18-AQ material (Dr. Maisch, GmbH) using two sequential gradients of 2 and 4 hours with increasing buffer B concentration and a 250 nl/min flow rate. The Q Exactive plus instrument (Thermo Fisher Scientific) was operated in the data dependent mode with a full scan in the Orbitrap followed by top 10 MS/MS scans using higher-energy collision dissociation (HCD). Settings for MS analysis is as follows: one full scan (resolution 70,000; m/z 300–1,700; target value 3e6; maximum injection 20 ms) followed by top 10 MS/MS scans (resolution 17,500; target value 1e6; maximum injection 60 ms) using higher-energy collisional dissociation (HCD) (isolation width, 2; normalized collision energy, 26). Ions with an unassigned charge state and singly charged ions were rejected. Former target ions selected for MS/MS were dynamically excluded for 30 s. MaxQuant software (v1.5.1.2)[3,4] was used to identify and quantify proteins. False discovery rate was set to 1% at both peptide and protein amount.

Data normalization for mass spectrometry and RNA-seq analyses

Similarly to other omics localization studies[5-9], we used generally accepted way of normalizing omics data that is considered the best practice: it presumes that the majority of genes are equally represented in two compared samples and the median fold change equals 1[10-12]. In case of proteomics analysis, for example, the LFQ (label-free quantification) normalizes two samples based on common peptides that are present in both neurites and soma samples, determines pair-wise ratios of peptide intensity for individual proteins, and then normalize the samples so that median ratio from pair-wise protein ratios is 1:1[13]. This approach enables identification of genes that are overrepresented (*e.g.* shown in *green* on MA plot **Fig 1D**) or underrepresented (*blue*) in the analyzed sample compared with the bulk of genes (grey), *i.e.* are above or below the general trend. Note that even if normalization is done on a different assumption (for example based on cell number), the median bulk of genes (*grey*) will shift along the Y axis, but the set of over- or underrepresented genes will stay the same.

The current way of data normalization has been applied by prior studies that compared gene expression between cell bodies and protrusions[5-9]. Normalization based on cell number has been generally avoided, because cell protrusions are far smaller than soma (3-20 fold in different studies depending on the test system used) and direct comparison of protrusions and soma on a per cell basis is not possible: such approach would rather measure the size of correspondent cell compartment, than differential gene expression. ERCC spike-ins were used to allow for the

absolute quantification of the RNA-seq data (**Supplementary Data 2**).

LFQ analysis of local proteome of iNeurons

All raw data were analyzed and processed with MaxQuant version 1.5.1.2[14]. Default settings were kept except that 'match between runs' was turned on. Search parameters included two missed cleavage sites, cysteine carbamidymethyl fixed modification, and variable modifications including methionine oxidation, protein N-terminal acetylation and deamidation of asparagine and glutamine. The peptide mass tolerance was 6 ppm and the MS/MS tolerance was 20 ppm. Database search was performed with Andromeda[15] against UniProt mouse database (downloaded on 2014-10) with common serum contaminants and enzyme sequences. False discovery rate (FDR) was set to 1% at peptide and at protein level.

In total, we identified 9057 proteins in neurites and soma of iNeurons. Proteins identified by only modified peptides, reverse hits and contaminants were filtered out using Perseus software6. Only proteins that were quantified in at least 2 replicates of neurites and/or soma sample were used for further analysis. To reduce the impact of mis-quantification due to the small number of detected peptides, the minimum ratio count was set to 2. Missing protein measurements were imputed by sampling from the normal distribution using Perseus software with default setting of the width of the Gaussian distribution relative to the standard deviation of measured values: 0.3, the amount by which the distribution used for the random numbers is shifted downwards: 1.86[16]. Differential expression analysis of proteomics data was done using two sample t-test with Perseus on normalized LFQ values. Using this approach, we quantified 7323 proteins. Proteins were designated as differentially expressed if the adjusted p value was lower than 0.05, and the absolute log2 fold change was greater than 1. As the assignment of peptides to genes is ambiguous with a gene possibly represented by more than one peptide group, a unique group was selected for each gene based on the number of detected peptides.

SILAC and pSILAC experiments

MaxQuant analysis was done as mentioned above. For SILAC experiments, Arg10/Lys8 were set as variable modifications in addition to those mentioned above. Minimum ratio count was set to 2. Re-quantification and match-between-runs were tuned on. For pSILAC experiments, Arg6/Lys4 and Arg10/Lys8 were set as variable modifications. Minimum ratio count was set to 1. Match-between-runs was activated.

QuaNCAT experiment

Raw files acquired in the QuaNCAT experiment were analyzed using MaxQuant ver.1.5.1.2[14]. The Andromeda search engine[15] matched the acquired MS/MS spectra to by trypsin/P *in silico* digested Uniprot mouse database (2014-10) and a database containing common contaminants. False discovery rate was estimated by in parallel searching reversed versions of the databases and set to 1% at both peptide and protein level. N-terminal acetylation, methionine oxidation and deamidation of asparagine and glutamine was set as variable modifications and cysteine carbamidomethylation was set as a fixed modification. "Re-quantify" and "match between runs" were activated. After filtering for "reverse hits", "potential contaminants" and "proteins only identified by site" we retained 1212 identified proteins. We further filtered the data using only proteins quantified with at least 3 SILAC counts and showed reproducibility between the label swap experiments as estimated by a smaller than 2 fold change in ratios between the reverse and forward experiment. We used the MaxQuant normalized values for the comparison with the Ribo-seq data and arrived at a set of 380 proteins, which showed a significant correlation with the Ribo-seq data (Pearson correlation coefficient 0.62).

RNA sequencing (RNA-seq) analysis

Raw reads were trimmed to remove adapters and low quality bases with the BBduk2 trimmer (https://sourceforge.net/projects/bbmap/): rref=$adapter k=10 threads=12 ktrim=r qtrim=r minlength=100 minoverlap=9 trimq=25. Cleaned reads were subsequently aligned using the STAR aligner[17] on the mm9 version of the mouse genome with the following parameters: --outFilterMultimapNmax 5 --outFilterMismatchNoverLmax 0.05. Reads were assigned to transcript models in a strand specific fashion using the SummarizeOverlaps function from Bioconductor[18]. Each gene was represented by a transcript model containing the highest number of exons. If two transcripts had the same number of exons, we chose the longer one.

RNA localization data from prior studies presented in **Supplementary Data 5** was obtained as follows. Data for hippocampal neurons was extracted form the Supplementary Table S2 of Cajigas *et al.*[19]. A log2 Neurite/Soma ratio was calculated for Nanostring (S2-Sheet 05) and RNA sequencing data (S2-Sheet 01, S2-Sheet 03), and merged into the **Supplementary Data 5** using provided MGI gene symbols. Data for cortical neurons, N2A and CAD neuronal cell lines, presented in Taliaferro *et al.*[5], was downloaded from the ENA SRA (SRP057123), and mapped to

the mm9 version of the mouse genome using STAR (with above-mentioned parameters). The expression was estimated as described above, using the summarizeOverlaps from Bioconductor, on a selected set of transcripts.

Ribosome profiling (Ribo-seq) analysis

Raw reads were trimmed to remove adapters and low quality bases with the the BBduk2 trimmer (https://sourceforge.net/projects/bbmap/): rref=$adapter_right. lref=$adapter_left k=10 ktrim=r qtrim=r minlength=10 minoverlap=5 trimq=25
Cleaned reads were subsequently aligned using the STAR aligner[17] on the mm9 version of the mouse genome with the following parameters: --outFilterMultimapNmax 5 --outFilterMismatchNoverLmax 0.05. To visualize the signal periodicity of the Ribo-seq data, we plotted the reads corresponding to the three translation frames, in a window of +/- 50-75 bp around the translation start site. If a transcript contained multiple translation start sites, the most upstream translation start site was chosen. Reads of width 28 and 29 nt were the most abundant. Reads were summarized over coding sequences. Differential expression analysis was done using DESeq2 as described in RNA sequence analysis. RPKM expression estimates were obtained by scaling the DESeq2 normalized counts by the corresponding CDS widths.

Differential expression analysis

Normalization and differential expression analysis was done using DESeq2[20] for all combined RNA-seq and Ribo-seq. To adjust for the different sizes of respective regions used for counting, transcript and CDS widths were used as normalization factors during size factor estimation. The procedure was performed with the independent.filtering parameter set to FALSE. Genes were designated as differentially expressed, if the adjusted p-value was < 0.05, and the absolute log2 fold change was > 1. RPKM expression estimates were obtained by scaling the DESeq2 normalized counts by the corresponding transcript/CDS widths.

CircRNA Analysis

Circular splice junctions were detected using Chiflex (Filipchyk et al., manuscript in preparation). Briefly, reads were aligned with Bowtie2 in local mode. All the reads mapped continuously to a genome were removed from further analysis. Others were tested to cover splice junctions. We applied the following criteria: read must map to

two different loci on the same chromosome with a distance less than 800 thousands base pairs, mappings must carry canonical splicing signals at their edges, the gap between two alignments on a read must be less than 2 nt and the overlap must be less than 5 nt. Moreover, an extensive filtering step was applied to further remove potential false positives. Reads were filtered according to their alignment score and positions of the mappings on the read. Exact thresholds for this filter were adjusted by Logic Rule Generator (manuscript in preparation) on the basis of read length, nucleotide composition and structure of mapping reference. Finally reads covering the same splice junction were merged and annotated to be linear (tail to head) or circular (head to tail).

CircRNA expression was quantified by counting the number of backsplicing reads over circularized exon junctions. CircRNAs were removed if they contained less than 5 backspliced reads in all samples. The expression of the linear isoform was quantified by counting the number of linearly spliced reads over the same corresponding donor and acceptor splice sites. Data was normalized and the log fold changes were estimated using DESeq2.

Data integration and functional classification of genes according to their localization patterns

To evaluate the statistical significance of the linear models we used the p-value of the F-statistic implemented in the R software. Using the RNA-seq data, neurite-localized proteome (proteomics neurites/soma log2FC > 1, p.values < 0.05) was split into 3 categories:

1. "Localized Protein and mRNA": mRNA enriched in neurites by at least 2-fold (RNA-seq neurites/soma log2FC > 1, p.values < 0.05)
2. "Localized Protein and moderately enriched mRNA": 0 < RNA-seq log2 neurites/soma < 1
3. "Localized Protein, but not mRNA": RNA-seq log2 neurites/soma < 0

Mean Ribo-seq data are shown for each category.

Additionally, we used two gene sets as for comparison in methylation sites enrichment analysis:

4. "Somatically localized genes": mRNA and protein log2FC neurites/soma < -1
5. "Equally distributed genes": -0.58 < mRNA and protein log2FC neurites/soma < 0.58

GO term over-representation analysis

GO term enrichment analysis was done using PANTHER (protein annotation through evolutionary relationship, release 20160321) Overrepresentation Test[21], with default settings. Overrepresentation analysis was performed on gene sets corresponding to different localization categories and genes overexpressed in iNeurons comparing with mESC. The exact parameters for gene set selection are indicated in the legends. For GO term over-representation analysis for RBPs localized to neurites (log2FC > 1, p-values < 0.05), we used the gProfileR package[22]. As a background set we considered all the RBPs detected using mass spectrometry in our study. The parameters for gProfileR were: correction_method = "fdr", organism = "mmusculus", hier_filtering = "moderate". In addition, we only consider results with a false discovery rate less than 0.025.

Motif enrichment analysis

For *de novo* motif identification, we used the MEME program[23] with the following parameter values: –rna, -minw 5, -mod zoops, -maxsize 20000000, -nmotifs 5, -evt 0.01 and a first-order Markov model as background. We searched the 5' and 3'UTRs of protein-coding mRNAs whose levels were higher in neurites compared to somas in both RNA-seq and proteomics datasets (neurites/soma log2FC > 1, p-values < 0.05). We located and counted MEME-identified motifs with the MAST software[24] using a cut-off expectation value (-ev) of 1 for motifs of 20 bp or shorter and 0.1 for larger motifs. As a reference set, we used soma-enriched protein-coding RNAs (RNA-seq and proteomics neurites/soma log2FC < -1, p.values < 0.05) or equally distributed RNAs (-0.58 < mRNA and protein log2FC neurites/soma < 0.58). Differential enrichment significance (neurites/soma) was assessed using Fisher's exact test. We only considered motifs with odds ratio greater than 2.0, p-value less than 5×10^{-3} and coverage of at least 5%. Coverage was defined as the percentage of significant motifs relative to the total number of sequences in its corresponding dataset. The odds ratio was used as a measure of enrichment. The odds ratio is defined as (a/b)/(c/d), a: number of sequences with significant MAST hits in test set, b: number of sequence with no significant hits in the test set, c: number of sequences with significant MAST hits in the reference set, and d: number of sequences with no significant hits in the reference set. The p-value of the odds ratio is indicated. The offset identifies the Tomtom alignment position relative to the MEME motif. The adj.p-value refers to the Tomtom alignment as well. In addition, we explored different maximum length values of MEME (10,15, 20, and 25). We chose the motif with the best odds ratio in each case. All the UTR sequences were retrieved from Ensemble

BioMart[25] using the BiomaRt interface[26]. When a 5'UTR or 3'UTR sequence was not available, the flanking sequence was used, 130 or 800 nt, respectively. These length values correspond to the median sequence length for each dataset. We compared the differentially-enriched motifs using the Tomtom software[27] against a database of RNA-binding motifs[28]. Pearson's correlation coefficient was used as the similarity metric. We used R software[29] for the statistical analysis.

Epitranscriptome (m^1A and m^6A) analysis

Experimentally obtained m1A and m6A positions in mouse liver, MEF, mES cells, amd mouse brain are extracted from respective publications[30,31]. These regions were overlapped with localized mRNA classes and background sets. Their statistical enrichment was obtained by comparing the observed frequency of overlaps between each of the localization sets and m1A/m6A experiments to the distribution of overlap frequencies obtained by random sampling. To construct the background distribution, 1000 protein-coding genes were sampled at random and overlapped with each of the experimental sets, and the procedure was repeated 1000 times. The enrichment was visualized using ComplexHeatmap R library (https://bioconductor.org/packages/release/bioc/html/ComplexHeatmap.html).

Supplementary references

1 Kaech, S. & Banker, G. Culturing hippocampal neurons. *Nat Protoc* **1**, 2406-2415, doi:10.1038/nprot.2006.356 (2006).
2 Rappsilber, J., Mann, M. & Ishihama, Y. Protocol for micro-purification, enrichment, pre-fractionation and storage of peptides for proteomics using StageTips. *Nat Protoc* **2**, 1896-1906, doi:10.1038/nprot.2007.261 (2007).
3 Cox, J. & Mann, M. MaxQuant enables high peptide identification rates, individualized p.p.b.-range mass accuracies and proteome-wide protein quantification. *Nat Biotechnol* **26**, 1367-1372, doi:10.1038/nbt.1511 (2008).
4 Luber, C. A. *et al.* Quantitative proteomics reveals subset-specific viral recognition in dendritic cells. *Immunity* **32**, 279-289, doi:10.1016/j.immuni.2010.01.013 (2010).
5 Taliaferro, J. M. *et al.* Distal Alternative Last Exons Localize mRNAs to Neural Projections. *Mol Cell*, doi:10.1016/j.molcel.2016.01.020 (2016).
6 Mardakheh, F. K. *et al.* Global Analysis of mRNA, Translation, and Protein Localization: Local Translation Is a Key Regulator of Cell Protrusions. *Dev Cell* **35**, 344-357, doi:10.1016/j.devcel.2015.10.005 (2015).
7 Mili, S., Moissoglu, K. & Macara, I. G. Genome-wide screen reveals APC-associated RNAs enriched in cell protrusions. *Nature* **453**, 115-119, doi:10.1038/nature06888 (2008).
8 Pertz, O. C. *et al.* Spatial mapping of the neurite and soma proteomes reveals a functional Cdc42/Rac regulatory network. *Proc Natl Acad Sci U S A* **105**, 1931-1936, doi:10.1073/pnas.0706545105 (2008).
9 Poon, M. M., Choi, S. H., Jamieson, C. A., Geschwind, D. H. & Martin, K. C. Identification of process-localized mRNAs from cultured rodent hippocampal neurons. *J Neurosci* **26**, 13390-13399, doi:10.1523/JNEUROSCI.3432-06.2006 (2006).
10 Anders, S. & Huber, W. Differential expression analysis for sequence count data. *Genome Biol* **11**, R106, doi:10.1186/gb-2010-11-10-r106 (2010).
11 Dillies, M. A. *et al.* A comprehensive evaluation of normalization methods for Illumina high-throughput RNA sequencing data analysis. *Brief Bioinform* **14**, 671-683, doi:10.1093/bib/bbs046 (2013).
12 Evans, C., Hardin, J. & Stoebel, D. M. Selecting between-sample RNA-Seq normalization methods from the perspective of their assumptions. *Brief Bioinform*, doi:10.1093/bib/bbx008 (2017).
13 Cox, J. *et al.* Accurate proteome-wide label-free quantification by delayed normalization and maximal peptide ratio extraction, termed MaxLFQ. *Mol Cell Proteomics* **13**, 2513-2526, doi:10.1074/mcp.M113.031591 (2014).
14 Cox, J. *et al.* A practical guide to the MaxQuant computational platform for SILAC-based quantitative proteomics. *Nat Protoc* **4**, 698-705, doi:nprot.2009.36 [pii] 10.1038/nprot.2009.36 (2009).
15 Cox, J. *et al.* Andromeda: a peptide search engine integrated into the MaxQuant environment. *J Proteome Res* **10**, 1794-1805, doi:10.1021/pr101065j (2011).
16 Tyanova, S. *et al.* The Perseus computational platform for comprehensive analysis of (prote)omics data. *Nat Methods*, doi:10.1038/nmeth.3901 (2016).
17 Dobin, A. *et al.* STAR: ultrafast universal RNA-seq aligner. *Bioinformatics* **29**, 15-21, doi:10.1093/bioinformatics/bts635 (2013).
18 Huber, W. *et al.* Orchestrating high-throughput genomic analysis with Bioconductor. *Nat Methods* **12**, 115-121, doi:10.1038/nmeth.3252 (2015).
19 Cajigas, I. J. *et al.* The local transcriptome in the synaptic neuropil revealed by deep sequencing and high-resolution imaging. *Neuron* **74**, 453-466, doi:10.1016/j.neuron.2012.02.036 (2012).

20 Love, M. I., Huber, W. & Anders, S. Moderated estimation of fold change and dispersion for RNA-seq data with DESeq2. *Genome Biol* **15**, 550, doi:10.1186/s13059-014-0550-8 (2014).
21 Mi, H., Muruganujan, A., Casagrande, J. T. & Thomas, P. D. Large-scale gene function analysis with the PANTHER classification system. *Nat Protoc* **8**, 1551-1566, doi:10.1038/nprot.2013.092 (2013).
22 Reimand, J. *et al.* g:Profiler-a web server for functional interpretation of gene lists (2016 update). *Nucleic Acids Res* **44**, W83-89, doi:10.1093/nar/gkw199 (2016).
23 Bailey, T. L. & Elkan, C. Fitting a mixture model by expectation maximization to discover motifs in biopolymers. *Proc Int Conf Intell Syst Mol Biol* **2**, 28-36 (1994).
24 Bailey, T. L. & Gribskov, M. Combining evidence using p-values: application to sequence homology searches. *Bioinformatics* **14**, 48-54 (1998).
25 Kinsella, R. J. *et al.* Ensembl BioMarts: a hub for data retrieval across taxonomic space. *Database (Oxford)* **2011**, bar030, doi:10.1093/database/bar030 (2011).
26 Durinck, S., Spellman, P. T., Birney, E. & Huber, W. Mapping identifiers for the integration of genomic datasets with the R/Bioconductor package biomaRt. *Nat Protoc* **4**, 1184-1191, doi:10.1038/nprot.2009.97 (2009).
27 Gupta, S., Stamatoyannopoulos, J. A., Bailey, T. L. & Noble, W. S. Quantifying similarity between motifs. *Genome Biol* **8**, R24, doi:10.1186/gb-2007-8-2-r24 (2007).
28 Ray, D. *et al.* A compendium of RNA-binding motifs for decoding gene regulation. *Nature* **499**, 172-177, doi:10.1038/nature12311 (2013).
29 R_Core_Team. R: A language and environment for statistical computing. *R Foundation for Statistical Computing, Vienna, Austria* (2016).
30 Dominissini, D. *et al.* The dynamic N(1)-methyladenosine methylome in eukaryotic messenger RNA. *Nature* **530**, 441-446, doi:10.1038/nature16998 (2016).
31 Meyer, K. D. *et al.* Comprehensive analysis of mRNA methylation reveals enrichment in 3' UTRs and near stop codons. *Cell* **149**, 1635-1646, doi:10.1016/j.cell.2012.05.003 (2012).
